# ﻿Revision of the Oriental species of the hoverfly genus *Paramixogaster* Brunetti, 1923 (Diptera, Syrphidae, Microdontinae)

**DOI:** 10.3897/zookeys.1208.122829

**Published:** 2024-07-25

**Authors:** Menno Reemer, Hariharakrishnan Sankararaman

**Affiliations:** 1 Naturalis Biodiversity Center, P.O. Box 9517, 2300 RA Leiden, Netherlands Naturalis Biodiversity Center Leiden Netherlands; 2 Department of Crop Protection (Entomology), Vanavarayar Institute of Agriculture, Manakkadavu, Pollachi, Coimbatore, Tamil Nadu 642103, India Department of Crop Protection (Entomology), Vanavarayar Institute of Agriculture Coimbatore India

**Keywords:** Ant flies, identification key, Indomalayan realm, morphology, new species, new synonyms, taxonomy

## Abstract

The species of the hoverfly genus *Paramixogaster* Brunetti, 1923 from the Oriental Region are revised. The resulting number of valid species is 15, of which the following four are described as new: *P.halmaherensis* Reemer, **sp. nov.**, *P.jubata* Reemer, **sp. nov.**, *P.kodaiana* Sankararaman & Reemer, **sp. nov.**, and *P.sulawesiana* Reemer, **sp. nov.** Three new synonymies are established: *Paramicrodondecipiens* de Meijere, 1917, **syn. nov.** is a junior synonym of *Microdonvespiformis* de Meijere, 1908; *Paramixogasterwegneri* Keiser, 1964, **syn. nov.** is a junior synonym of *Ceratophyaindica* Doleschall, 1857; *Microdonsubpetiolatus* Thompson, 2020, **syn. nov.** is a junior synonym of *Microdoncontractus* Brunetti, 1923. *Paramixogasterhuoi* Reemer, **nom. nov.** is introduced as a replacement name for *P.trifasciatus* Huo & Zhao, 2022, which is a primary homonym of *P.trifasciatus* Ssymank & Reemer, 2016. Neotypes are designated for *Paramixogastericariiformis* Pendlebury, 1927 and *Myxogastervariegata* Sack, 1922, and a lectotype is designated for *Microdonvespiformis* de Meijere, 1908. An identification key to the species and diagnoses for all species are provided.

## ﻿Introduction

**Figures 1–3.**
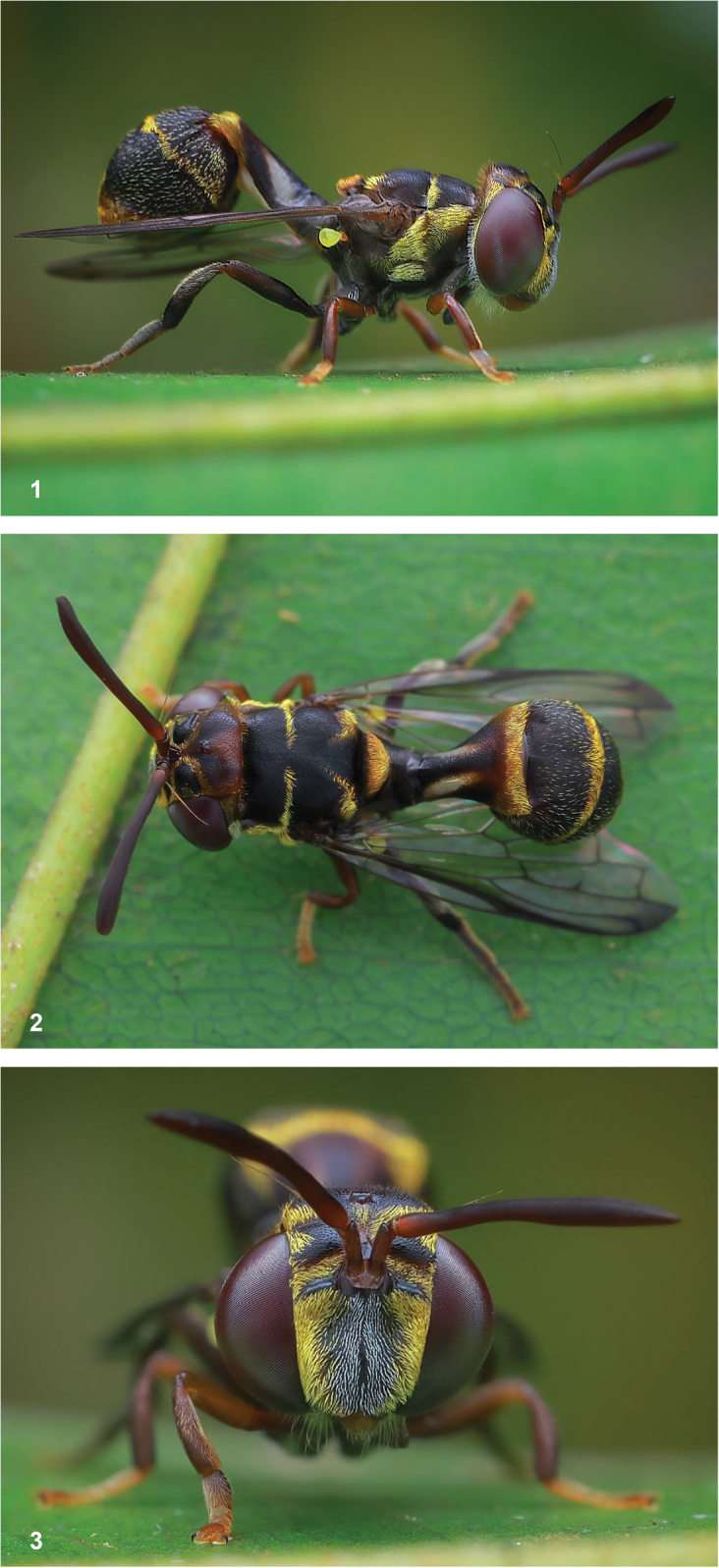
Hoverflies of the genus *Paramixogaster* are slender wasp mimics, with a more or less constricted abdomen and long antennae. This male of an undescribed species was photographed in Terengganu, Malaysia, on 26 June 2021. This specimen is very similar to *P.huoi* Reemer, nom. nov. and *P.sulawesiana* Reemer, sp. nov., from which it differs by the medially interrupted fascia of golden setulae along the transverse suture on the scutum (continuous in the other two species), as well as by the dark hind legs (yellowish in the other two species). It is also similar to *P.kodaiana* sp. nov., but differs in the less extensive infuscation of the wings and the medially interrupted golden fascia along the posterior margin of the scutum (continuous in *P.kodaiana* Sankararaman & Reemer, sp. nov.). No collected specimens of this species are known, which is why it is not described in this paper. Photographs by Husni Che Ngah.

**Figure 4.**
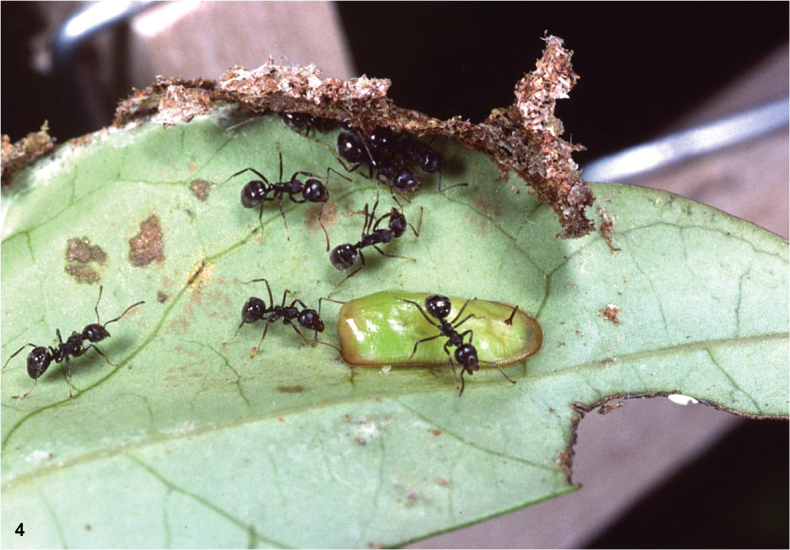
Larva of *Paramixogastervespiformis* (de Meijere), attended by ants, putatively of *Dolichoderusthoracicus* (Smith). Found in Thailand and reared to the adult stage by Greg R. Ballmer. For further details, see species account of *P.vespiformis*. Photograph by G.R. Ballmer.

Hoverflies of the genus *Paramixogaster* Brunetti, 1923 are slender wasp mimics, with a more or less constricted abdomen and long antennae (Figs 1–3). They are found in the Afrotropical, Oriental, and Australasian Regions (Reemer and Ståhls 2013a). Like most other Microdontinae, but unlike most other Syrphidae, flies of this genus are not known to visit flowers (Reemer 2012). Larvae of most species are unknown, but records of a few African and Australasian species have been found in nests of ants belonging to the subfamilies Formicinae and Myrmicinae (Hymenoptera: Formicidae) (Reemer 2013). A larva of the Oriental species *Paramixogastervespiformis* (de Meijere, 1908) was found in association with a species of the subfamily Dolichoderinae (Fig. 4; see species account of *P.vespiformis*).

*Paramixogaster* was erected by Brunetti (1923) after he recognised that *Mixogastervespiformis* Brunetti, 1913, a species he described from India ten years earlier, did not fit well into the New World genus *Mixogaster* Macquart, 1842. During the 20^th^ century, only two more species were included in *Paramixogaster*: *P.icariiformis* Pendlebury, 1927 from Peninsular Malaysia and *P.wegneri* Keiser, 1964 from Ambon (Indonesia, Moluccas). The latter species has been included in species catalogues of both the Oriental and the Australasian Region (Knutson et al. 1975; Thompson and Vockeroth 1989).

When Cheng and Thompson (2008) argued that *Paramixogasteroides* Shiraki, 1930 and *Tanaopicera* Hull, 1945 should be considered synonymous with *Paramixogaster*, the type species of both genus group names were also included in *Paramixogaster*: the Oriental *Myxogastervariegata* Sack, 1922 and the Australian *Ceratophyavariegata* Walker, 1852, respectively. Huang and Cheng (2012) described two *Paramixogaster* species from southern China. So, by that time, seven species were included in *Paramixogaster*, i.e., five from the Oriental Region and two from the Australasian Region, namely *P.variegatus* (Walker) from Australia and *P.wegneri* from Ambon.

The concept of *Paramixogaster* as a genus was renewed by Reemer and Ståhls (2013a, b), based on the analysis of morphological and molecular characters. They included 26 species in the genus, most of which were placed in other genera during the 19^th^ and 20^th^ century. According to the classification of Reemer and Ståhls (2013a), the genus is distributed not only in the Oriental and Australasian Regions, but also in the Afrotropical Region.

Since the introduction of the new generic concept by Reemer and Ståhls (2013a, b), two additional *Paramixogaster* species were described: *P.trifasciatus* Ssymank & Reemer, 2016 from Central African Republic and *P.trifasciatus* Huo & Zhao in Zhao & Huo, 2022 from China. The latter is a primary homonym of the former and we provide a replacement name in this work.

This paper revises the Oriental species of *Paramixogaster*. Before starting this revision, 13 Oriental species were included in this genus (Reemer and Ståhls 2013a; Zhao et al. 2022). These species are revised here, introducing new synonymies as well as new species, resulting in 15 Oriental species in the genus.

## ﻿Materials and methods

Morphological terminology largely follows Cumming and Wood (2017) (for wing venation the ‘traditional’ system is used), supplemented with some terms specifically introduced for Microdontinae by Reemer and Ståhls (2013b).

Type material of previously described species has been studied when available to us. The types of *Paramixogastericariiformis* Pendlebury, 1927, *P.fujianensis* Cheng, 2012, and *P.yunnanensis* Cheng, 2012 could not be studied (for details see species accounts). For these species, we had to rely on the original descriptions.

For studied primary types, text on labels is given ad verbatim. Text is indicated in quotation marks (“ ”) and each line on the label is separated by a double forward slash (//). Text not given on labels (i.e., remarks by authors) is given in square brackets ([]).

Collections are indicated by the following acronyms:

**CASB**Chinese Academy of Sciences, Beijing (China)

**DEI**Senckenberg Deutsches Entomologisches Institut, Müncheberg (Germany)

**NHMUK**Natural History Museum, London (United Kingdom)

**NMB**Naturhistorisches Museum Basel, Basel (Switzerland)

**NBAIR** National Bureau of Agricultural Insect Resources, Bengaluru (India)

**QSBG** Queen Sirikit Botanical Gardens, Chiang Mai (Thailand)

**RMNH**Naturalis Biodiversity Center, Leiden (the Netherlands)

**SUHC** Shaanxi University of Technology, Hanzhong (China)

**UCRC**University of California, Dep. of Entomology, Riverside (USA)

**USNM**United States National Museum, Smithsonian Institution, Washington D.C. (USA)

**ZMHU**Museum für Naturkunde, Berlin (Germany)

**ZMUC**Natural History Museum of Denmark, Copenhagen (Denmark)

**ZSI**Zoological Survey of India, Calcutta (India)

Photographs have been made over the course of 15 years using various types of equipment across a range of circumstances in a number of different institutions, the details of which could no longer be retrieved.

## ﻿Taxonomic account

### ﻿ Paramixogaster

Taxon classificationAnimaliaDipteraSyrphidae

Brunetti, 1923

C72A8086-5737-5180-815D-396C23816ACA

#### Generic diagnosis.

Among Microdontinae, *Paramixogaster* is one of two microdontine genera which combines a bare postpronotum with a constricted abdomen. The only other genus to which this combination of characters applies is the Neotropical genus *Ceriomicrodon* Hull, 1937. This taxon differs from *Paramixogaster* in the widely rounded postero-apical corner of wing cell r_4+5_ (angular in *Paramixogaster*), and in the long and whip-like dorsal process of the phallus (short and as long as ventral process in *Paramixogaster*).

In two African species, the abdomen is not clearly constricted in dorsal view, but tergite 2 is dorsoventrally flattened, making the abdomen appear constricted in lateral view (Reemer and Ståhls 2013a). There are no such species known from the Oriental Region.

#### Nomenclatural note.

As stated by ICZN article 30.1.2, names ending in -*gaster* are feminine, so the genus name *Paramixogaster* will be treated as such in this paper. Thus, conventional Latin adjectives are treated as adjectives by default under the Code (1999, Article 31.2). Nevertheless, nouns do not need to agree in gender with the generic name (ICZN, 1999, Article 31.2.1) and species-group names that can be regarded as a noun or as an adjective, when the author did not indicate how to treat them, are treated as a noun in apposition and the original spelling is to be retained (ICZN 1999, Article 31.2.2).

### ﻿Key to Oriental species of *Paramixogaster*

Note that several of the included species are known from one sex only, so sexual dimorphism is unknown for these species. In general, it seems that *Paramixogaster* females tend to have a shorter postpedicel than males, as well as a larger body size. As in many other Syrphidae, females also differ from males in a wider face and abdomen. Also note that the type specimens of *P.fujianensis* and *P.yunnanensis* have not been examined, so their placement in this key is based on the descriptions and therefore should be considered tentative.

**Table:** 

1	Postpronotum bare, abdomen constricted basally. Postero-apical corner of wing cell r4+5 angular	**2 (*Paramixogaster*)**
–	Other combination of characters	other Microdontinae genera
2	Frons not clearly swollen, without lateral bulges (Fig. 5)	**10**
–	Frons swollen, with pair of lateral bulges, with narrow sulcus in between (Fig. 6)	**3**
3	Tergite 2 > 2× as long as wide (Huang and Cheng 2012: fig. 415)	***P.fujianensis* Cheng**
–	Tergite 2 < 2× as long as wide (Figs 17, 18, 23, 99)	**4**
4	Mesoscutum without golden setulae along transverse suture, at most with a small patch of golden setulae laterally at notopleuron (Fig. 7)	**8**
–	Mesoscutum with fascia of golden setulae along transverse suture, either continuous or medially interrupted (Fig. 8)	**5**
5	Tergite 2 with lateral margins strongly and abruptly curved upward posteriorly, giving it a saddle-like appearance (Fig. 9). Tergites 3 and 4 without fasciae of golden setulae along posterior margins (Fig. 68)	***P.icariiformis* Pendlebury**
–	Tergite 2 with lateral margins only slightly and evenly curved upward posteriorly (Fig. 10). Tergites 3 and 4 with wide fasciae of dense golden setulae along posterior margins (Figs 60, 81, 102)	**6**
6	Fascia of golden setulae along transverse suture on scutum medially interrupted (Fig. 86). Wing clearly infuscate in anterior cells, with dark colouration extending into wing cell R4+5 (Fig. 87). Tergites 3 and 4 with short black setulae over most of their surface (except for the golden posterior fasciae)	***P.kodaiana* Sankararaman & Reemer, sp. nov.^1^**
–	Fascia of golden setulae along transverse suture on scutum continuous (Fig. 60). Wing slightly brownish in anterior cells, but less clearly and dark colouration not extending into cell R4+5 (Figs 66, 106). Tergites 3 and 4 with pale (yellowish, with golden intermixed) setulae over most of their surface (except for the golden posterior fasciae)	**7**
7	Apex of vein R2+3 at approximately same level as joint M1 with R4+5 (Fig. 11). Face entirely yellow (Fig. 63)	***P.huoi* Reemer, nom. nov.**
–	Apex of vein R2+3 more distal than joint of M1 with R4+5 (Fig. 12). Face dark medially (Fig. 105)	***P.sulawesiana* Reemer, sp. nov.**
8	Tergite 2 (except for yellow maculae) reddish at least on apical 1/2 (Fig. 30). Mesoscutum reddish with a median black vitta of ~ 1/3 the width of the scutum (Fig. 31)	***P.brunettii* Reemer**
–	Tergite 2 (except for yellow maculae) black, at most narrowly reddish along posterior margin (Figs 54, 55). Mesoscutum dark brown to blackish, at most with pale colouration along margin (Fig. 58)	**9**
9	Tergite 2 with posterior margin narrowly reddish. Postalar callus dark. Wing entirely clear. Male: Postpedicel 8× as long as scape	***P.yunnanensis* Cheng**
–	Tergite 2 with posterior margin entirely dark brown (Fig. 55). Postalar callus yellow. Wing with infuscate apical 1/2 of cells r1 and r2+3 (Fig. 59). Male: Postpedicel 6× as long as scape	***P.halmaherensis* Reemer, sp. nov.**
10	Postpedicel shorter than scape (Fig. 13)	***P.luxor* (Curran)**
–	Postpedicel longer than scape (Fig. 14)	**11**
11	Transverse suture incomplete, medially interrupted. Metanepisternum bare. Tergite 2 with pair of elongate yellow maculae, which are either entirely separated or connected anteriorly (Figs 23, 24)	**13**
–	Transverse suture complete, medially not interrupted. Metanepisternum setulose. Tergite 2 with wide, continuous yellow fascia (Figs 17, 18)	**12**
12	Femora entirely yellow. Vein M1 recurrent at more or less right angle (Fig. 15). Tergite 2 constricted less strongly (Fig. 17). Wing cell r4+5 bare at basal 1/4 to 1/3	***P.contracta* (Brunetti)**
–	Femora partly black. Vein M1 recurrent at acute angle (Fig. 16). Tergite 2 constricted more strongly (Fig. 18). Wing cell r4+5 entirely microtrichose	***P.conveniens* (Brunetti)**
13	Tergites 3 and 4 yellow with pattern of black vittae (Fig. 95). Mesoscutum largely yellow, with two small black maculae posterior to postpronotum and two elongate narrow black maculae between transverse suture and posterior margin (Fig. 21)	***P.sacki* Reemer & Ståhls**
–	Tergites 3 and 4 black with yellow posterior margin (Fig. 74, 118, 123). Mesoscutum usually largely black, often with narrow or wide yellow margins, sometimes also with yellow median line and yellow fascia along transverse suture (Figs 22, 73)	**14**
14	Tergite 2 at posterior margin narrower than median length of tergite (Fig. 23). Vertex with setulae at least twice as long as diameter of ocelli (Fig. 25). Scutellum entirely yellow (Fig. 23). Katatergite entirely dark. Male genitalia: surstylus baso-ventrally with a hook-like process (Fig. 135)	***P.jubata* Reemer, sp. nov.**
–	Tergite 2 at posterior margin wider than median length of tergite (Fig. 24). Vertex with setulae approximately as long as diameter of ocelli (Fig. 26). Scutellum with anterior margin dark (Fig. 123). Katatergite yellow with dark macula posteriorly. Male genitalia: surstylus baso-ventrally rounded (Figs 136, 137)	**15**
15	Mesoscutum with lateral yellow vitta continuous from postpronotum to postalar callus, although often narrower posteriad of transverse suture (Fig. 27). Male: postpedicel 4.4–5.6× as long as scape. Male genitalia as in Fig. 136	***P.indica* (Doleschall)**
–	Mesoscutum with lateral yellow vitta interrupted posteriad of transverse suture (Fig. 28). Male: postpedicel 3.3–3.7× as long as scape. Male genitalia as in Fig. 137	***P.vespiformis* (de Meijere)**

**Figures 5, 6.**
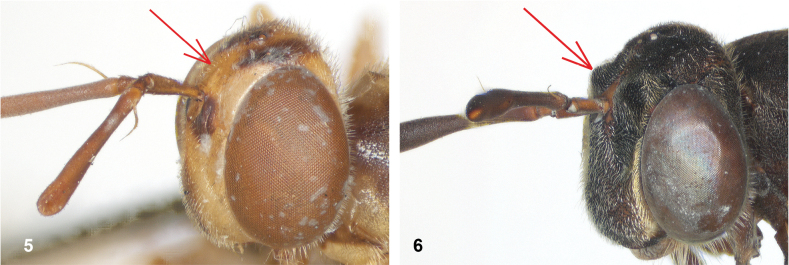
Head, dorsolateral: **5** frons more or less even, without lateral bulges (*Paramixogastervespiformis* female, Indonesia) **6** frons uneven, with lateral bulges (*P.icariiformis* female, neotype).

**Figures 7, 8.**
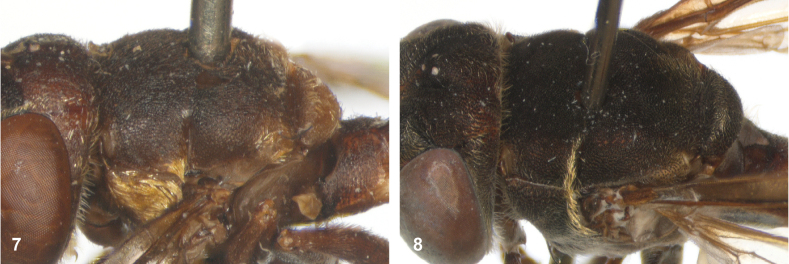
Scutum, dorsolateral: **7** transverse suture without fascia of golden setulae (*Paramixogasterhalmaherensis* Reemer, sp. nov. male, holotype) **8** transverse suture with fascia of golden setulae (*P.icariiformis* female, neotype).

**Figures 9, 10.**
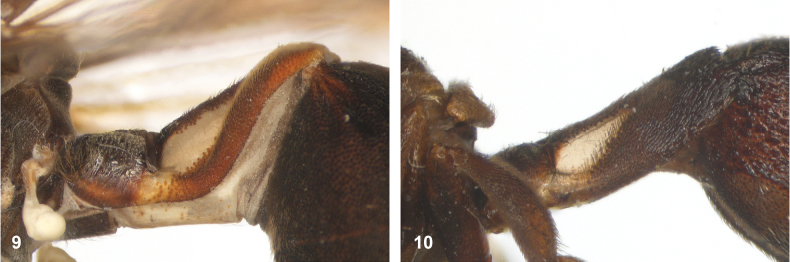
Tergite 2, lateral: **9***Paramixogastericariiformis* female, neotype **10***P.halmaherensis* Reemer, sp. nov. male, holotype.

**Figures 11, 12.**
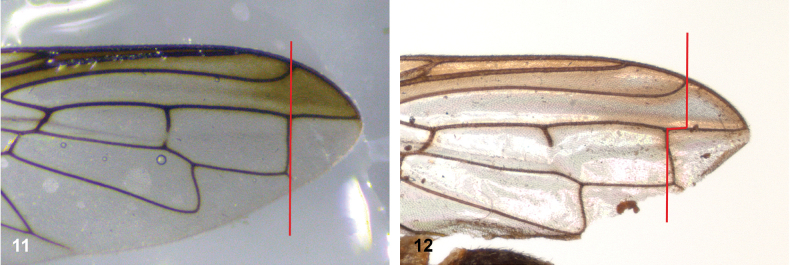
Position of apex of vein R_2+3_: **11** at approximately same level as joint M_1_ with R_4+5_ (*Paramixogasterhuoi* Reemer, nom. nov. holotype) **12** more distal than joint of M_1_ with R_4+5_ (*P.sulawesiana* Reemer, sp. nov. holotype).

**Figures 13, 14.**
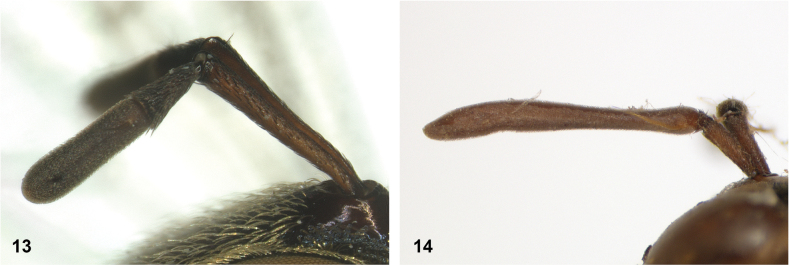
Ratio of antennal segments: **13** postpedicel shorter than scape (Paramixogastercf.luxor female, Sabah) **14** postpedicel longer than scape (*P.vespiformis* male, Sumatra).

**Figures 17, 18.**
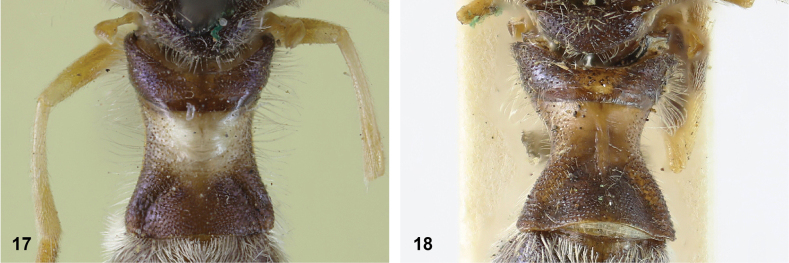
Tergite 2 dorsal: **17***Paramixogastercontracta* female, holotype **18***P.conveniens* female, holotype.

**Figures 15, 16.**
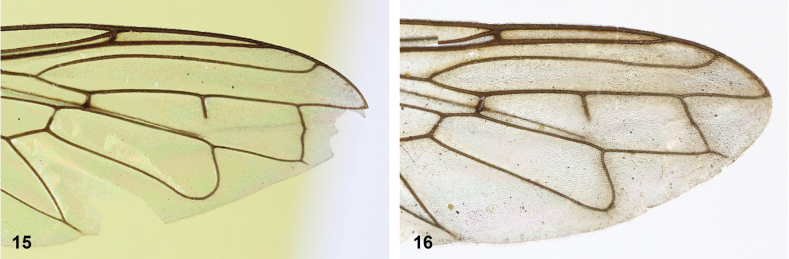
Apex of wing: **15** vein M_1_ recurrent at more or less right angle with R_4+5_ (*Paramixogastercontracta* holotype) **16** vein M_1_ recurrent at acute angle (*P.conveniens* holotype).

**Figures 19, 20.**
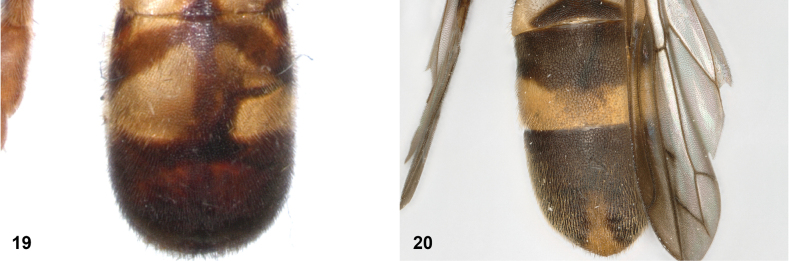
Tergites 3 and 4 dorsal: **19***Paramixogastersacki* male, Taiwan **20***P.indica* male, Ambon.

**Figures 21, 22.**
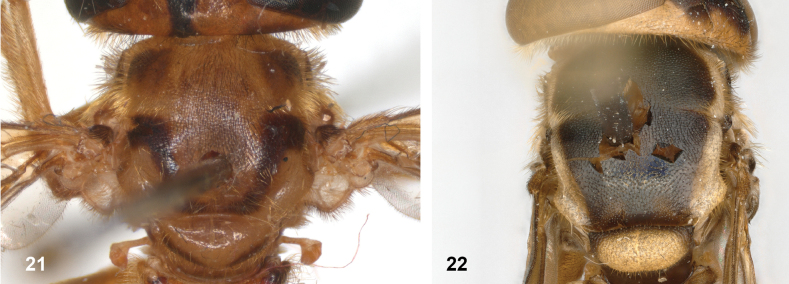
Mesoscutum dorsal: **21** largely yellow with two four black maculae (*Paramixogastersacki* male, Taiwan) **22** largely black with yellow margins (*P.indica* male, Ambon).

**Figures 23, 24.**
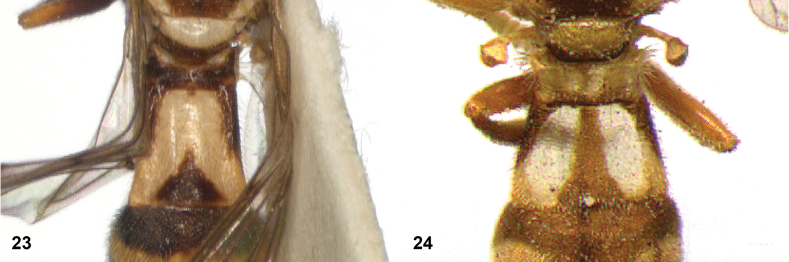
Tergite 2 dorsal: **23** posterior margin narrower than median length of tergite (*Paramixogasterjubata* Reemer, sp. nov. male, holotype) **24** posterior margin wider than median length of tergite (*P.vespiformis* (de Meijere), lectotype).

**Figures 25, 26.**
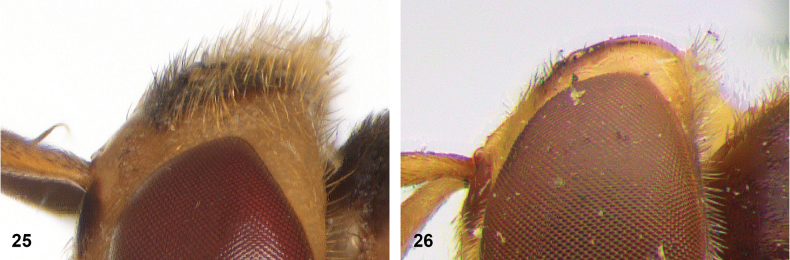
Vertex lateral: **25** setulae at least twice as long as diameter of ocelli (*Paramixogasterjubata* Reemer, sp. nov. male, holotype) **26** setulae approximately as long as diameter of ocelli (*P.vespiformis* female, Java).

**Figures 27, 28.**
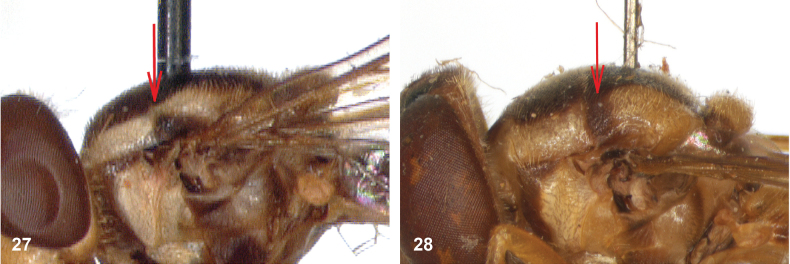
Mesoscutum lateral: **27** lateral yellow vitta continuous from postpronotum to postalar callus (*Paramixogasterindica*) **28** lateral yellow vitta interrupted posteriad of transverse suture (*P.vespiformis*).

### ﻿Species accounts

### ﻿ Paramixogaster brunettii

Taxon classificationAnimaliaDipteraSyrphidae

Reemer in Reemer & Ståhls, 2013

D91A88F3-18DF-511C-AD08-FA7DDB9EB48D

Figs 29–31

Mixogaster vespiformis Brunetti, 1913: 169. Holotype ♂: India (ZSI) [photographs studied] (secondary homonym of Microdonvespiformis de Meijere, 1908, see Reemer and Ståhls 2013a).
Paramixogaster vespiformis (Brunetti) – Brunetti 1923: 320; Knutson et al. 1975: 373.
Paramixogaster brunettii Reemer, 2013 in Reemer & Ståhls, 2013a: 144.

#### Studied type specimens.

***Holotype*** of *Mixogastervespiformis* Brunetti, 1913. India • 1 ♂; N.E. Assam, Dibrugarh; 17–19 Nov. 1911; ZSI. Label 1: “Ind. Mus. // Dibrugarh // N.E. Assam // Abor Exped // 17–19-XI-11. // Kemp [printed vertically on left side of label]”; label 2: “Mixogaster // vespiformis // Brun. Typ. ♀”; label 3: “Paramixogas- // ter. // vespiformis // Brun Type ♀ // det. Brun. 1923”; label 4: “2177 // [illegible 2 digit code]” [digital images studied].

#### Diagnosis.

Only male known. Body length: 8 mm. A reddish-brown species with lateral bulges on the frons. From most other species with lateral bulges on the frons, it differs by the combination of the following characters: tergite 2 less than twice as long as wide, mesoscutum without fascia of golden setulae along transverse suture. This species is most similar to *P.halmaherensis* and *P.yunnanensis*, but differs because the mesoscutum is reddish with a median black vitta of ~ 1/3 of the width of the scutum (mostly dark brown to blackish in the other two species), and tergite 2 is reddish at least on the apical 1/2 (at most narrowly reddish along posterior margin in the other two species).

#### Notes.

*Mixogastervespiformis* Brunetti, 1913 was included in *Paramixogaster* by Brunetti (1923). The species name became a secondary homonym when *Microdonvespiformis* de Meijere, 1908 was transferred to *Paramixogaster* by Reemer and Ståhls (2013a), who introduced the replacement name *Paramixogasterbrunettii* Reemer, 2013. The type specimen itself has not been studied by the authors, but digital images were kindly provided by Jeroen van Steenis.

#### Distribution.

Only known from Assam (India).

### ﻿ Paramixogaster contracta

Taxon classificationAnimaliaDipteraSyrphidae

(Brunetti)

ADF2DCD1-6D74-561F-8D04-2098B1AE2F76

Figs 15 , 17 , 32–38 , 119–122 , 114–116 , 130

Microdon contractus Brunetti, 1923: 310. Holotype ♀: India (NHMUK) [examined]; Knutson et al. 1975: 370.
Paramixogaster contractus (Brunetti) – Reemer and Ståhls 2013a: 145.
Microdon subpetiolatus Thompson, 2020: 87. Syn. nov., comb. nov. Holotype ♂: Sri Lanka, Ratnapura District, Singharaja Forest (USNM) [examined].

#### Studied type specimens.

***Holotype*** of *Microdoncontractus* Brunetti, 1923. India • 1 ♀; Deesa; 1922; C.G. Nursee leg.; NHMUK. Label 1 (round, red-bordered): “Holotype”; label 2: “Deesa // P.T.O // 3. 97.”; label 3: “India: // Pres. by // Col. C.G. Nurse. // 1922 309.”; label 4: “Microdon // contractus // Brun Type [female] // det. Brun. 1921”.

***Holotype*** of *Microdonsubpetiolatus* Thompson, 2020. Sri Lanka • 1 ♂; coll. USNM. Label 1: “SRI LANKA: Rat. Dist. // Singharaja forest // 5 VIII 1973 600 ft. // Ginter Ekis”; label 2: “collected // in Malaise // trap”; label 3 (large, orange): “Holotype // Microdon // subpetiolatus // Thompson ‘19”; label 4 (with QR-code): “USNMENT // 01541885”.

***Paratype*** of *Microdonsubpetiolatus* Thompson, 2020. Sri Lanka • 1 ♂; coll. USNM. Label 1: “SRI LANKA: Tri. Dist. // Trincomalee, China // Bay Ridge Bungalow // 0–100’, 13–17-V-1976”; label 2: “Collected by // K.V. Krombein // P.B. Karunaratne // S. Karunaratne // D.W. Balasooriya”; label 3: “Microdon // 08-03 // det. // FC Thompson 19”; label 4 (yellow): “Paratype // Microdon // subpetiolatus // FCT-2010”; label 5 (with QR-code): “USNMENT // 01541884”.

#### Diagnosis.

Body length: male 7.5 mm, female 9 mm. This species and *P.conveniens* share a unique character among Microdontinae: a setulose metanepisternum. Within *Paramixogaster*, both species also stand out because of the complete transverse suture on the mesoscutum. *Paramixogastercontracta* differs from *P.conveniens* in the following characters: legs entirely yellow (femora partly black in *P.conveniens*) and vein M_1_ recurrent at more or less right angle (acute angle in *P.conveniens*). Male genitalia as in Fig. 130.

#### Redescription

**(based on holotype of *M.contractus* Brunetti). Adult female.** Body length: 9 mm.

***Head*.** Face occupies 45% of head width in frontal view, with sides below antennae parallel; black; entirely white setulose. Gena widely developed; black; white setulose. Oral margin not notched anteriorly, laterally not produced. Frons and vertex blackish brown; white setulose. Ocellar triangle not elevated. Occiput black; white setulose. Eye very sparse and short setulose, almost bare. Antennal fossa approximately as high as wide. Antenna dark brown, scape paler; postpedicel ~ 1.25× as long as scape. Postpedicel parallel-sided with rounded apex; with small sensory pit at apical 1/3. Arista slender, ~ 1/2 as long as postpedicel.

***Thorax*.** Mesoscutum shining blackish brown; entirely white setulose. Postpronotum pale brown; bare. Postalar callus pale brown; white setulose. Scutellum shining blackish brown; white setulose. Anepisternum with shallow sulcus separating posterior from anterior part; blackish; white setulose, with small bare part ventro-medially. Anepimeron blackish; entirely white setulose. Katepisternum blackish brown; white setulose dorsally; bare ventrally. Katatergite pale brown; long microtrichose. Anatergite pale brown; short microtrichose. Calypter and halter yellow.

Wing: hyaline; microtrichose, except bare on cells bc and c, basal 3/5 of cell r_1_, basal 1/4 of cell r_2+3_, basal 1/3 of cell r_4+5_, antero-basal 1/6 of cell dm, entirely on cells br and bm, basal 2/3 of cell cup, and medially on alula.

Legs: Pale yellow; yellow to white setulose.

***Abdomen*.** Tergite 2 constricted and tergites 3 and 4 widened; narrowest point at anterior 1/4 of tergite 2, widest at posterior margin of tergite 3. Tergites blackish brown, except anterior 1/2 of tergite 2 pale yellow; white setulose, except with short brown setulae on posterior 1/2 of tergite 2 and posterior 2/3 of tergite 3, with white setulae long and conspicuous on anterior 1/3 of tergite 3 and anterior 1/2 of tergites 4 and 5. Tergite 2 with median tubercle on anterior 1/3. Sternites pale brown; yellow setulose.

**Male** (based on holotype and paratype of *M.subpetiolatus* Thompson).

As female, except for following differences. Body length: 7.5 mm. Face occupies 42–46% (*n* = 2) of head width in frontal view. Postpedicel 1.2–1.4× as long as scape (*n* = 2).

#### Notes.

The type specimen of *Microdoncontractus* Brunetti (a female) (Figs 32–38) was compared to those of *M.subpetiolatus* Thompson (male holotype and male paratype: Figs 109–116). They were found to be very similar, except for some differences usually associated with sexual dimorphism, such as a slightly wider abdomen in the female. No differences were found that could support specific taxonomic status for both taxa, so they are here considered as synonyms, with *M.subpetiolatus* Thompson syn. nov. as the junior synonym.

#### Distribution.

Known from India (Gujarat: Deesa) and Sri Lanka.

### ﻿ Paramixogaster conveniens

Taxon classificationAnimaliaDipteraSyrphidae

(Brunetti, 1923)

B738C48E-0653-50CD-B3B3-C941279B0713

Figs 39–45

Microdon conveniens Brunetti, 1923: 311. Holotype ♀: India (NHMUK) [examined]; Knutson et al. 1975: 370.
Paramixogaster conveniens (Brunetti) – Reemer and Ståhls 2013a: 145.

#### Studied type specimens.

***Holotype*** of *Microdonconveniens* Brunetti. India • 1 ♀; Assam; Cameron leg.; NHMUK. Label 1 (round, red-bordered): “Holotype”; label 2: “Assam.”; label 3: “Cameron Coll. // 1902-105”; label 4: “M. conveniens // Brun. Type. [female] // det. Brun. 1923”.

#### Diagnosis.

Only female known. Body length: 9.5 mm. This species and *P.contracta* share a unique character among Microdontinae: a setulose metanepisternum. Within *Paramixogaster*, both species also stand out because of the complete transverse suture on the mesoscutum. *Paramixogasterconveniens* differs from *P.contracta* in the following characters: legs with femora partly black (entirely yellow in *P.contracta*) and vein M1 recurrent at acute angle (more or less right angle in *P.contracta*).

#### Redescription

**(based on holotype). Adult female** Body length: 9.5 mm.

***Head*.** Face occupying ~ 1/3 of head width in frontal view, with sides more or less parallel below antennae; black; entirely white setulose. Gena widely developed; black; white setulose. Oral margin not notched anteriorly, laterally not produced. Frons and vertex black; yellowish white setulose. Ocellar triangle not elevated. Occiput black; white setulose. Eye bare. Antennal fossa approximately as high as wide. Antenna: scape brown [other segments are missing in type specimen].

***Thorax*.** Mesoscutum shining black, brownish along margins; entirely white setulose. Postpronotum pale brown; bare. Postalar callus pale brown; white setulose. Scutellum shining blackish brown; white setulose. Anepisternum with shallow sulcus separating posterior from anterior part; blackish brown; white setulose, except bare on narrow strip along anterior margin. Anepimeron blackish brown; entirely white setulose. Katepisternum blackish brown; white setulose dorsally; bare ventrally. Katatergite brown; long microtrichose. Anatergite brown; short microtrichose. Calypter and halter brownish yellow.

Wing: hyaline; microtrichose, except bare on cell bc and basal 1/3 of cell c, basal 1/4 of cell r_1_, basal 2/3 of cell br, basal 1/2 of cell bm, basal 1/2 of cell cup, and baso-median 1/2 of alula.

Legs: Pale yellow, except hind femur dark brown on basal 3/4 and hind tibia with brown ring on apical 1/3; yellow to white setulose.

***Abdomen*.** With tergite 2 constricted and tergites 3 and 4 widened; narrowest point at anterior 1/4 of tergite 2, widest at posterior margin of tergite 3. Tergites blackish brown, except anterior 1/2 of tergite 2 yellow; entirely white setulose, with setulae long and conspicuous on anterior 1/4 of tergite 3, anterior 1/3 of tergite 4 and on most of tergite 5. Sternites brown, white setulose.

#### Distribution.

Only known from Assam (India).

### ﻿ Paramixogaster fujianensis

Taxon classificationAnimaliaDipteraSyrphidae

Cheng in Huang & Cheng, 2012

38D01B8A-4A9F-51F7-A5F0-7358A57224C7

Paramixogaster fujianensis Cheng in Huang & Cheng, 2012: 695. Holotype ♂: China, Yunnan (CASB, but see notes) [not examined]; Reemer and Ståhls 2013a: 145.

#### Diagnosis.

Only male known. Body length: male 12 mm. Among the species with lateral bulges on the frons (which may not be as clear in this species as in other ones), *P.fujianensis* is the only one in which tergite 2 is more than twice as long as wide (Huang and Cheng 2012: fig. 415). The mesonotal transverse suture is incomplete. According to the English translation of the description, the postpedicel is 3× as long as the scape (Huang and Cheng 2012). Figures of habitus and head are provided by Huang and Cheng (2012). Note that these characters are based on the description only and could not be verified against any specimens.

#### Notes.

Unsuccessful attempts were made to locate the type specimen of *Paramixogasterfujianensis* by trying to contact the author and by enquiring at the CASB collection (Ke-Ke Huo pers. comm. 2023). The original description by Huang and Cheng (2012) is in Chinese, but the same work also provides an English translation, as well as figures of the head in frontal view and of the thorax and abdomen in dorsal view. This information makes clear that this species is distinct from all other known Oriental species of *Paramixogaster*, especially in the very elongate tergite 2.

### ﻿ Paramixogaster halmaherensis

Taxon classificationAnimaliaDipteraSyrphidae

Reemer sp. nov.

2CFD4CFE-577F-54C7-B841-4111C30F358C

https://zoobank.org/00A93B1B-3A27-4B8B-A79F-B87A1E70B955

Figs 54–59 , 131

#### Type material.

***Holotype*.** Indonesia • 1 ♂; Halmahera, Dodinga (sealevel); 2–4 Nov. 1951; coll. RMNH. Label 1: “Isl. halmaheira // Dodinga (sealevel) // 2,4-XI-1951 // Native Collector”; label 2: “Mus. Zool. Bogor // Microdon ves- // piformis de Meijere // Det. Adisoemarto”; label 3 (red): “HOLOTYPE // Paramixogaster // halmaherensis // Reemer 2024”.

#### Diagnosis.

Only male known. Body length: 8 mm. This is one of the three species with a swollen frons with lateral bulges (Figs 56, 57) and without golden setulae along the transverse suture on the scutum (Fig. 58) (the other two are *P.brunettii* and *P.yunnanensis*). From *P.brunettii* it differs because tergite 2 is black (reddish in *P.brunettii*) with a pair of yellow maculae. From *P.yunnanensis* it differs by the yellow postalar calli (dark in *P.yunnanensis*), postpedicel 6× as long as scape (8× as long as scape in *P.yunnanensis*), and the infuscate apical 1/2 of wing cells r_1_ and r_2+3_ (wing entirely clear in *P.yunnanensis*). Male genitalia as in Fig. 131.

#### Description

**(based on holotype). Adult male.** Body length: 8 mm.

***Head*.** Face occupying ~ 1/2 of head width in frontal view, with sides somewhat converging ventrad; brown with widely yellow lateral and ventral margins; golden yellow setulose except for narrow median bare line. Gena yellow; yellow setulose. Oral margin not notched anteriorly, laterally weakly produced. Frons posteriorly with blackish pair of lateral bulges which are short black setulose, separated by narrow pale brown crease; anteriorly with pair of slightly concave areas which are yellow setulose, separated from face by pair of shiny black bare maculae. Vertex swollen; brown; short black setulose. Occiput yellow, somewhat darker dorsally and ventrally; white setulose. Eye bare. Antennal fossa approximately as high as wide. Antenna orange-brown; postpedicel ~ 6× as long as scape. Arista slender, yellow, a little longer than the scape.

***Thorax*.** Mesoscutum blackish brown with lateral and posterior margins somewhat paler; short black setulose, except for small patch of golden yellow setulae anterior to notopleuron and large patch of pale yellowish setulae anterior to scutellum. Postpronotum pale brown, bare. Postalar callus yellow, yellow setulose. Scutellum without calcars; yellow; yellow setulose. Pleura yellowish brown. Anepisternum entirely covered with thick golden yellow setulae, appressed and directed hindward. Katepisternum long golden yellow setulose dorsally; bare ventrally. Katepimeron with a few long yellow setulae. Katatergite and anatergite short microtrichose. Metanotum shining brown. Calypter greyish yellow. Halter yellow.

Wing: hyaline; microtrichose, except bare in cell r_1_ narrowly along Rs, basal 1/3 of r_4+5_, entirely on br (except for microtrichia along vena spuria), antero-basal 1/4 of dm, entirely on bm, basal 2/3 of cup.

Legs: yellowish brown, except mid-femora and hind-legs darker brown; yellow setulose.

***Abdomen*.** Constricted basally, narrowest at transition between tergites 1 and 2, widest at transition between tergites 3 and 4. Tergite 1 dark brown; short white setulose, except long black setulose at antero-lateral callus. Tergite 2 dark brown with two large, elongate, pale yellow maculae from anterior margin to ~ 1/2 of tergite; short black setulose, except bare on yellow maculae and narrowly yellow setulose along lateral margin. Tergite 3 dark brown; black setulose except for triangular patch of white setulae at postero-lateral corners. Tergite 4 dark brown; short black setulose with sparse longer golden yellow setulae intermixed and with patch of white setulae at antero-lateral corners. Sternite 1 brown; short black setulose. Sternite 2 yellow; bare. Sternites 3 and 4 brown; short black setulose. Genitalia as in Fig. 131.

#### Distribution.

Only known from Halmahera (Indonesia).

#### Etymology.

The specific epithet is to be treated as a noun and refers to the type locality.

### ﻿ Paramixogaster huoi

Taxon classificationAnimaliaDipteraSyrphidae

Reemer nom. nov.

97BA43CF-8F37-5BAF-8321-6F8383389AF5

Figs 11 , 60–66

Paramixogaster trifasciatus Huo & Zhao in Zhao & Huo, 2022: 4; primary homonym of Paramixogastertrifasciatus Ssymank & Reemer, 2016: 404.

#### Studied type specimens.

***Holotype*** of *Paramixogastertrifasciatus* Huo & Zhao. CHINA • 1 ♂, Guangdong, Shenzhen City, Wutong Mountains; 114°21'E, 22°57'N; 927 m above sea level; 25 April 2020; Zuqi Mai leg.; coll. SUHC [only photos studied].

#### Diagnosis.

Only male known. Body length: 7 mm. It belongs to the group of species with lateral bulges on the frons (Figs 63, 64). From *P.fujianensis* it differs by tergite 2 being < 2× as long as wide (> 2× as long as wide in *P.fujianensis*). From *P.icariiformis* it differs by the presence of fasciae of golden setulae along the posterior margins of tergites 3 and 4 (absent in *P.icariiformis*). There is a continuous fascia of golden setulae along the mesoscutal transverse suture, and also along the posterior margin of the scutum, and there are fasciae of golden setulae along the posterior margins of tergites 3 and 4 (Figs 60–62). These fasciae are not as dense and sharply demarcated as in *P.kodaiana* Sankararaman & Reemer, sp. nov., but more similar to those in *P.sulawesiana* Reemer, sp. nov. From the latter species, *P.huoi* Reemer, nom. nov. differs by the colouration of the face, which is yellow with a vaguely darker median vitta (mostly dark with lateral margins yellow in *P.sulawesiana* Reemer, sp. nov.), and the wing venation: the apex of R_2+3_ is situated at approximately the same level as the joint of M_1_ with R_4+5_. (more distal than joint of M_1_ with R_4+5_ in *P.sulawesiana* Reemer, sp. nov.).

#### Notes.

The name *Paramixogastertrifasciatus* was already used by Ssymank and Reemer (2016) for an African species. When Huo and Zhao (in Zhao and Huo 2022) described a new species from China under the same name, a primary homonym was created. As a replacement name, *Paramixogasterhuoi* Reemer, nom. nov. is proposed here, in honour of Ke-Ke Huo, one of the original authors, who was kind enough to provide photographs of the holotype.

### ﻿ Paramixogaster icariiformis

Taxon classificationAnimaliaDipteraSyrphidae

Pendlebury, 1927

4DAC136B-C0F0-59CF-8C59-17BAC2DFBFF6

Figs 6 , 8 , 9 , 67–72

Paramixogaster icariiformis Pendlebury, 1927: 38. Holotype ♀: Malaysia, Selangor, Bukit Kuta (lost); Knutson et al. 1975: 373; Reemer and Ståhls 2013a: 145.

#### Studied type specimens.

***Neotype*** of *Paramixogastericariiformis* Pendlebury (new neotype designation, see notes). Thailand • 1 ♀; Loei, Phu Ruea NP Sa Sawan, 17°30.735'N, 101°20.601'E, alt. 1352 m asl., 12–10 March 2007; leg. Patikhom Tumtpip; coll. QSBG. Label 1: “THAILAND Loei, Phu Ruea NP Sa Sawan, // 17°30.735'N, 101°20.601'E, 1352 m, // Malaise trap, 12–19.iii.2007, Patikhom // Turntip leg. T2309”; label 2: “MR315 // DNA-voucher // Y1076”; label 3 (red): “NEOTYPE // *Paramixogaster* // *icariiformis* Pendlebury, 1927 // Designated by Reemer & // Sankararaman 2024.

#### Diagnosis.

Only female known. Body length: 11 mm. This is the only known Oriental species of *Paramixogaster* that is entirely black except for tergite 2 red (Figs 67, 68). Another unique character of this species is the ‘saddle-like’ shape of tergite 2, due to its lateral margins being strongly curved upward posteriorly (Figs 9, 72).

#### Redescription

**(based on neotype). Adult female.** Body size: 11 mm.

***Head*.** Face at level of antennae occupying ~ 0.6 of head width in frontal view, with sides quite strongly converging ventrad; brown, more blackish in dorsolateral depressed areas, narrowly pale yellow ventrally close to oral gena; white setulose medially, golden yellow setulose laterally. Gena black; white setulose. Frons with blackish pair of lateral bulges which are short golden yellow setulose, separated by narrow pale brown triangular depression. Vertex swollen; black; short black setulose medially, short yellow setulose laterally and posteriorly. Occiput black; yellow setulose dorsally, white setulose ventrally. Eye bare. Antennal fossa slightly wider than high. Antenna brown; postpedicel ~ 4× as long as scape. Arista slender, yellow, ~ 2× as long as scape.

***Thorax*.** Mesoscutum black; short black setulose, except for narrow fascia of golden yellow setulae along transverse suture of 1/5 of width of mesoscutum, and patch of yellowish setulae of approximately the size of the scutellum anterior to scutellum. Postpronotum brown, bare. Postalar callus yellow, yellow setulose. Scutellum without calcars; black; yellow setulose. Pleura dark brown. Anepisternum and anepimeron white setulose, except for narrow fascia of golden yellow setulae along postero-dorsal margin, which is connected to the fascia of golden yellow setulae along the transverse suture of the mesoscutum. Katepisternum long white setulose dorsally; bare ventrally. Katepimeron with a few long white setulae. Katatergite and anatergite short microtrichose. Metanotum brown. Calypter pale grey. Halter white.

Wing: infuscate anteriorly from costal vein to vena spuria and anterior 1/3 of cell r_4+5_; microtrichose, except bare in cell br posteriad of vena spuria, all of bm, postero-basal 1/4 of cell r_4+5_, antero-basal 1/4 of dm, most of cup except microtrichose in distal corner, most of alula except microtrichose along margins.

Legs: pale brown, except hind femur and hind tibia dark brown; white setulose.

***Abdomen*.** Constricted basally, narrowest at basal 1/4 of tergite 2, widest at around 1/2 of tergite 3. Tergite 1 dark brown; white setulose. Tergite 2 with lateral margins strongly curved upward posteriorly; orange-brown with two large, elongate, pale yellow maculae from anterior margin to just more than half of tergite; short golden yellow setulose, more densely so along posterior margin, with black setulae between yellow maculae and bare on these maculae. Tergite 3 black; very short black setulose except for longer whitish setulae sparsely intermixed laterally. Tergite 4 as tergite 3, except narrowly yellow laterally along posterior margin. Tergite 5 black with yellow lateral and posterior margins; black setulose on black parts, yellow setulose on yellow parts. Sternite 1 brown; sparsely yellow and black setulose. Sternite 2 whitish yellow; sparsely yellow and black setulose. Sternites 3–5 blackish; white setulose.

#### Notes.

The original type of this species is registered to be in the collection of the NHMUK (Evenhuis and Pape 2022), but it is not there (N. Wyatt pers. comm. 2022). Probably this type is lost. However, the description by Pendlebury (1927) agrees very well with the female specimen from Thailand here designated as neotype. According to Pendlebury (1927), this is an entirely black species except for the reddish tergite 2. There are not fasciae of yellow or golden setulae on the thorax or abdomen. No other known species of *Paramixogaster* have the same colouration (entirely black with red tergite 2).

#### Distribution.

Malaysia and Thailand.

### ﻿ Paramixogaster indica

Taxon classificationAnimaliaDipteraSyrphidae

(Doleschall, 1857)

AFACC0EC-E77C-5875-84F6-88E125CBD196

Figs 20 , 22 , 123–124 , 125–129 , 136

Ceratophya indica Doleschall, 1857: 404. Holotype: Indonesia, Ambon (HNHM, lost) [not examined].
Microdon indicus (Doleschall, 1857) – Thompson and Vockeroth 1989: 438.
Paramixogaster indicus (Doleschall, 1857) – Reemer and Ståhls 2013a: 145.
Paramixogaster wegneri Keiser, 1964: 84. Syn. nov. Holotype ♂: Indonesia, Maluku, Ambon (NMB) [examined]; Knutson et al. 1975: 374; Thompson and Vockeroth 1989: 439; Reemer and Ståhls 2013a: 145.

#### Studied type specimens.

***Holotype*** of *Paramixogasterwegneri* Keiser. Indonesia • 1 ♂; label 1 (red): “TYPUS”; label 2 (pale green): “INDONESIA // AMBON // 6.I.61 // A.M.R. WEGNER”; NMB.

***Paratype*** of of *Paramixogasterwegneri* Keiser. Indonesia • 1 ♀; Ambon; 1 Jan. 1961; A.M.R. Wegner leg.; NMB.

***Paratypes*** of of *Paramixogasterwegneri* Keiser. Indonesia • 5 ♂; Ambon; 5 different collection dates: 29 Oct. 1960, 23 Nov. 1960, 8 Dec. 1960, 12 Dec. 1960, 11 Jan. 1961; A.M.R. Wegner leg.; NMB.

#### Additional specimens.

Indonesia • 2 ♂ 2 ♀; Buru; 7 Dec. 1921; L.J. Toxopeus leg.; RMNH; 1 ♀; Buru; 8 Dec. 1921; L.J. Toxopeus leg.; RMNH • 1 ♀; Ambon [no further data]; NHMUK • 1 ♀; West Papua, Fak-Fak; A.E. Pratt leg.; NHMUK [013933418].

#### Diagnosis.

Body length: males 7.5–9.5 mm (*n* = 8), females 9–11 mm (*n* = 6). This belongs to the group of species without lateral bulges on the frons. From *P.luxor* it differs by the postpedicel being longer than the scape (shorter in *P.luxor*). From *P.contracta* and *P.conveniens* it differs by the incomplete transverse suture (complete in *P.contracta* and *P.conveniens*). From *P.sacki* it differs by tergites 3 and 4 being black with yellow posterior margin (yellow with pattern of black vittae in *P.sacki*). It differs from *P.jubata* Reemer, sp. nov. by the shorter tergite 2, of which the posterior margin is wider than the median length (longer in *P.jubata* Reemer, sp. nov.), the dark anterior margin of the scutellum (entirely yellow in *P.jubata* Reemer, sp. nov.) and the shorter setulae on the vertex.

*Paramixogasterindica* is most similar to *P.vespiformis*, from which it differs by the continuous yellow vitta between postpronotum and posterior callus (interrupted in *P.vespiformis*), and the longer postpedicel in the male, which is 4.4–5.6× as long as the scape (3.3–3.7× as long in *P.vespiformis*). Male: postpedicel 4.4–5.6× as long as scape (*n* = 6). Female: postpedicel 2.4–2.9× as long as scape (*n* = 5). The degree of infuscation of wing apex is very variable. Male genitalia as in Fig. 136.

#### Notes.

No type specimen of *Ceratophyaindica* Doleschall is known. However, the description and illustration of this species from Ambon by Doleschall (1857) correspond well to the studied type specimens of *Paramixogasterwegneri* by Keiser (1964), which was also described from Ambon. Keiser (1964) does not mention *P.indica*, so probably he was unaware of it. Based on the descriptions and illustrations, these taxa share the following characters: postpedicel 4–6× longer than scape, face, and vertex partly yellow, mesoscutum black, wing with infuscate apex, tergite 2 narrowest at base and with two apically diverging yellow vittae, tergites 3 and 4 black with yellow hind margins. Based on these similarities and the shared type locality (Ambon), *P.wegneri* Keiser syn. nov. is here considered to be a junior synonym of *Ceratophyaindica* Doleschall.

This species is very closely related to *P.vespiformis* (de Meijere). The differences between these taxa are small but consistent.

#### Distribution.

Known from eastern parts of Indonesia, east of the Wallace Line: the Maluku islands of Ambon and Buru, and West Papua. Specimens were collected between October and January.

### ﻿ Paramixogaster jubata

Taxon classificationAnimaliaDipteraSyrphidae

Reemer sp. nov.

1C684D56-EEDE-5496-ADAC-F30E9811F698

https://zoobank.org/DDEE3975-7CDC-4B9D-A6AB-59ED50BAB188

Figs 23 , 25 , 73–80 , 135

#### Type material.

***Holotype*.** Vietnam • 1 ♂, holotype of *Paramixogasterjubata* sp. nov. Reemer; S. Vietnam, Dông Nai, Cat Tien National Park; alt. 100 m.; 13–20 May 2007; C. van Achterberg & R. de Vries leg.; RMNH. Label 1: “S. Vietnam: Dông Nai // Cat Tien N.P., ca. 100 m // 13–20.v.2007. Mal. traps // 25–29, eco-trail; C. v. Achter- // berg & R. de Vries, RMNH’07”; label 2 (red): “HOLOTYPE // Paramixogaster // jubata // Reemer 2024”.

***Paratypes*.** Vietnam • 1 ♂; Cat Tien National Park; 13–20 May 2021; Malaise trap; C. van Achterberg & R. de Vries leg.; RMNH; MZH DNA voucher Y0721. [abdomen missing].

Thailand • 1 ♂; Phitsanulok T567, Thung Salaeng Luang National Park; 580 m.; 16°50.217'N, 101°52.541'E; 11–18 Aug. 2006; Malaise trap; Pongpilak Pranee leg.; QSBG.

#### Diagnosis.

Only male known. Body length: 6–7 mm. A small species without lateral bulges on the frons and postpedicel 3.3–3.7× as long as scape (*n* = 3). In colouration it looks much like *P.indica* and *P.vespiformis*, from which it differs by the longer than wide tergite 2 (wider than long in *P.indica* and *P.vespiformis*) and the long setulae on the vertex, which are at least twice as long as the diameter of the ocelli (approximately as long as diameter of ocelli in *P.indica* and *P.vespiformis*) (Figs 25, 76). Male genitalia as in Fig. 135.

#### Description

**(based on holotype). Adult male** Body size: 7 mm.

***Head*.** Face occupying ~ 2/5 of head width in frontal view, with sides slightly converging ventrad; yellow, except for brown macula laterad of antennal fossa and median brown macula of almost 1/3 of width of face; yellow setulose, except black setulose on median brown macula. Gena yellow, yellow setulose. Oral margin laterally hardly produced. Frons yellow; yellow setulose. Vertex with large median dark brown macula, yellow laterally; black setulose on brown part, yellow setulose on yellow parts. Occiput yellow, yellow setulose. Eye bare. Antennal fossa approximately as high as wide. Antenna with scape and pedicel yellow, postpedicel brown; postpedicel ~ 3.7× as long as scape. Arista slender, yellow, slightly longer than scape.

***Thorax*.** Mesoscutum black with yellow fascia along transverse suture and yellow median vitta from anterior margin to ~ 3/4 of length; mostly black setulose on black parts and yellow setulose on yellow parts, except for large median patch of longer yellow setulae on posterior 1/3. Postpronotum yellow, bare. Postalar callus yellow, yellow setulose. Scutellum without calcars; yellow; yellow setulose. Pleurae yellow, except anterior anepisternum, ventral part of posterior anepisternum, ventral katepisternum and katepimeron brown. Anepisternum with shallow sulcus separating posterior from anterior part; yellow setulose anteriorly and posteriorly, with wide bare part in between. Anepimeron entirely long yellow setulose. Katepisternum long yellow setulose dorsally; bare ventrally. Katatergite long microtrichose, anatergite short microtrichose. Metanotum shining brown. Calypter grey. Halter yellow.

Wing: hyaline; microtrichose, except bare on cell br (only with microtrichia along vena spuria), on basal 1/2 of cell bm, and basal 1/3 of cell cup.

Legs: yellow, except hind tibia and basal 2/3 of hind tibia brown; yellow setulose, except mid tibia, hind tibia, and hind femur black setulose. Coxae and trochanters dark brown.

***Abdomen*.** Constricted basally, narrowest at tergite 1, widest halfway tergite 4. Tergite 1 blackish brown; black and yellow setulose. Tergite 2 with two long, oblique, pale-yellow maculae from anterior to posterior margin, which leave blackish brown antero-lateral corners and a blackish brown posterior triangle; mostly yellow setulose, but with some yellow setulae anteriorly and yellow setulose along lateral margin. Tergite 3 blackish brown on slightly less than anterior half, yellow posteriorly; black setulose on dark parts, yellow setulose on yellow parts. Tergite 4 blackish brown on anterior 3/5, except for median yellow triangle, which is an extension of the yellow fascia on the posterior 2/5 of the tergite; black setulose on anterior 3/5, yellow setulose on posterior 2/5. Sternite 1 yellow; bare. Sternites 2 and 3 yellow; yellow setulose. Sternite 4 yellowish with vague dark brown markings; black setulose. Genitalia as in Fig. 135.

#### Distribution.

Thailand and Vietnam.

#### Etymology.

The specific epithet *jubata* (Latin, adjective) means ‘with manes’. This refers to the setulae on the vertex, which are longer than in the resembling species *P.indica*.

### ﻿ Paramixogaster kodaiana

Taxon classificationAnimaliaDipteraSyrphidae

Sankararaman & Reemer sp. nov.

9E90BDC3-E757-53BE-AE30-4160A397F3AF

https://zoobank.org/D7109D90-0F62-49EF-9877-DA7014DFF8D5

Figs 81–87

#### Type material.

***Holotype*.** India • 1 ♀; Vattakanal, Kodaikanal; 2070 m a.s.l.; 10°12'54.03"N, 77°29'07.15"E; 4 January 2022; H. Sankararaman leg.; NBAIR.

Label 1: “India: Vattakanal, Kodaikanal, 10°12'54.03"N, 77°29'07.15"E // Sankararaman. H leg. 04.i.2022"; label 2 (red): “*Paramixogasterkodaiana* sp. nov. // det. Sankararaman & Reemer”.

***Paratype*.** India • 1 ♀; Kodaikanal, Pulney Hills, S. India; 1980 m asl [6500 ft.]; April–May 1953; P.S. Nathan leg.; USNM [unique specimen identifier USNMENT01541882].

#### Diagnosis.

Only female known. Body length: 11 mm. *Paramixogasterkodaiana* Sankararaman & Reemer, sp. nov. belongs to the group of species with lateral bulges on the frons. From *P.fujianensis* it differs by terigte 2 being less than twice as long as wide (more than twice as long as wide in *P.fujianensis*). From *P.icariiformis* it differs by the presence of fasciae of golden setulae along the posterior margins of tergites 3 and 4 (absent in *P.icariiformis*). From *P.huoi* Reemer, nom. nov. and *P.sulawesiana* Reemer, sp. nov. it differs by the medially interrupted fascia of golden setulae along the transverse suture of the scutum (continuous in *P.huoi* Reemer, nom. nov. and *P.sulawesiana* Reemer, sp. nov.).

#### Description

**(based on holotype). Adult female.** Body length: 11 mm.

***Head*.** Face occupying less than 1/3 of head width in frontal view, with sides slightly converging ventrally; dark brown; golden setulose. Gena brown; golden setulose. Oral margin slightly produced laterally. Frons brown; golden setulose. Vertex brown; golden setulose. Ocellar triangle slightly elevated. Occiput wide dorso-laterally, narrowing down ventrally; brown; golden yellow setulose dorsally adjacent to vertical triangle, long silvery white setulose ventrally. Eye bare. Antennal fossa approximately as high as wide. Antenna mostly brown, scape and pedicel orange-brown, postpedicel black; ratio of lengths of scape, pedicel and postpedicel approximately as 1:0.4:4.4. Scape ~ 2.4× the length of pedicel, postpedicel 4.4× the length of scape. Arista orange-brown, slender, arising close to base of postpedicel and ~ 0.4× (2/5) of length of postpedicel.

***Thorax*.** Mesoscutum dark brown to black, golden setulose. Postpronotum black, bare, but with sparsely scattered long golden setulae, posteriorly. Mesonotum black with fine punctures; with fascia of dense golden setulae along the transverse suture (medially interrupted) and along posterior margin, including postalar calli. Scutellum without calcars; black; dense golden setulose. Pleura brown to black, with short white microtrichia. Anepisternum and katepisternum with golden setulae along posterior margin as extension of golden fascia along mesonotal transverse suture. Anepimeron short white setulose, other pleurites short brown to black microtrichia. Katepisternum blackish brown; long golden yellow setulose dorsally; bare ventrally. Katatergite brown to black; white microtrichose. Metanotum dark brown. Calypter yellow. Halter yellow.

Wing: microtrichose, except for bare regions in basal 1/5 of cells c, bc, br, almost entire bm, basal 2/3 of cup and medially on alula. Blackish antero-apically with distinct dark cloud (apical part of r_1_, entire r_2+3_, anterior part of r_4+5_) and brownish on basal 1/2, otherwise subhyaline. Stigmal crossvein present. Alula ~ 2× as long as wide.

Legs: brown to reddish brown, with silvery white setulae. Coxae and trochanters dark brown to black with pale setulae, hind coxa with golden setulae. Hind femur dark brown to black basally. Tibiae and tarsi reddish brown.

***Abdomen*.** Segment 2 constricted and segments 3 and 4 widened; narrowest point at posterior tergite 1, widest at posterior margin of tergite 3. Tergites dark brown to black with fine punctures as in mesonotum. Tergite 1 dark brown, with long, scattered, silvery white setulae. Tergite 2 black, constricted and dorsoventrally flattened, with widely separated yellow vittae, anteriorly wide and oblique, posteriorly narrowed down and parallel not reaching the golden setulae along the posterior margin of tergite 2. Dense golden setulae on tergite 2, 3, and 4 posteriorly, scattered golden microtrichia on tergites 3, 4 and 5 dorso-laterally. Sternite 2 pale brown, short yellow setulose. Other sternites brown.

**Male.** Unknown.

#### Notes.

This species is likely to mimic wasps belonging to the genus *Ropalidia* Guérin-Méneville, 1831 (Hymenoptera: Vespidae: Polistinae) by having similar colouration on wing and abdomen. The resting behavior was also very similar: the fly was perching on the underside of a leaf holding its abdomen upright with wings kept flat, as various species *Ropalidia* also do.

#### Habitat.

The holotype was collected in montane evergreen shola forests, in proximity to human settlement. The dense vegetation included several perennial trees, ferns, and grass. The fly was found perching on the leaves of guava (*Psidium* L.).

#### Distribution.

Only known from the type locality in Tamil Nadu, southern India.

#### Etymology.

This species is named after the type locality, Kodaikanal (nicknames ‘Princess of hill stations’), which is located in upper Palani hills of Tamil Nadu. The epithet is a noun in apposition.

### ﻿ Paramixogaster luxor

Taxon classificationAnimaliaDipteraSyrphidae

(Curran, 1931)

9DF9BBDF-6D2F-51A2-B3A6-2E12B17CB93F

Figs 13 , 88–90 , 91–94 , 132

Microdon luxor Curran, 1931: 306. Holotype ♂: Malaysia (NHMUK) [examined]; Knutson et al. 1975: 371.
Paramixogaster luxor (Curran, 1931) – Reemer and Ståhls 2013a: 145.

#### Studied type specimens.

***Holotype*** of *Microdonluxor* Curran. Malaysia • 1 ♂; Selangor, Bukit Kutu; 20 Apr. 1926; H.M. Pendlebury leg.; NHMUK. Label 1 (small, round, red-bordered): “Holo- / type”; label 2 (red): “Microdon / Type / luxor [male sign] / Curran / No.”; label 3: “Malay Penin: / Selangor, / Bukit Kutu / 3500 ft. / April 20^th^ 1926. / H.M. Pendlebury.”; label 4: “Pres. by / Fed. Malay States / Museum / B.M. 1934-74.”.

#### Additional specimens.

Malaysia (identification uncertain, see notes) • 1 ♀; SE Sabah, nr. Danum Valley Field C; Malaise trap 7; alt. 150 m.; 20–22 Nov. 1997; C. van Achterberg leg; RMNH (M. Reemer specimen code MR.

Thailand • 1 ♀; Songkhla, Nam Tok Ton Pliu; 17 Feb. 2005; Yanega, D. leg.; UCRC.

#### Diagnosis.

This is the only known Oriental species of *Paramixogaster* in which the scutellum has a pair of apical calcars, and also the only one in which the postpedicel is shorter than the scape. Male genitalia as in Fig. 132.

#### Redescription

**(based on holotype). Adult male** Body length: 7 mm.

***Head*.** Face occupying ~ 1/4 of head width in frontal view, with sides slightly converging ventrad; black, entirely yellow setulose. Gena narrow, black, yellow setulose. Oral margin laterally slightly produced. Frons and vertex black, yellow setulose. Ocellar triangle not elevated. Occiput black, yellow setulose. Eye bare. Antennal fossa approximately as high as wide. Antenna blackish, except scape brown on basal 4/5; antennal ratio approximately as 4:1:3. Arista slender, slightly more than half the length of postpedicel.

***Thorax*.** Mesoscutum black; short black setulose, except yellow setulose along posterior margin [probably also along transverse suture and possibly along anterior margin, but not visible in type specimen because of grease]. Postpronotum brown, bare. Postalar callus yellowish brown, yellow setulose. Scutellum black; black setulose; with two small apical calcars. Pleurae dark brown. Anepisternum without sulcus; entirely yellow setulose. Anepimeron entirely long yellow setulose. Katepisternum long yellow setulose dorsally; bare ventrally. Katatergite and anatergite short microtrichose. Calypter and halter yellow.

Wing: hyaline; microtrichose, except bare on cell bc, basal 1/3 of cell c, basally on cell r_1_ along vein Rs, entirely on cell br (only with microtrichia along vena spuria), on basal 2/3 of cell bm, antero-basal 1/3 of cell cup.

Legs: [Front legs missing in holotype]. Mid leg with femur dark brown on basal 2/3, yellow on apical 1/3; tibia and tarsus yellow; dark setulose on dark parts, yellow setulose on yellow parts. Hind leg with femur dark brown, tibia yellow on basal 2/5 and dark brown on apical 3/5, tarsus yellow; entirely yellow setulose. Coxae and trochanters dark brown; yellow setulose.

***Abdomen*.** Constricted, with narrowest point at anterior margin of tergite 2, widest point at posterior margin of tergite 3. Tergite 1 black; yellow setulose. Tergite 2 black with two large, yellow maculae on anterior 3/4; black setulose, except yellow setulose postero-laterally. Tergites 3 and 4 black [colour of setulae hard to assess in type specimen because of grease, but there seems to be oblique vittae of yellow setulae and a yellow setulose fascia along the posterior margin of tergite 4]. Sternite 1 black; bare. Sternite 2 yellow; yellow setulose. Sternites 3 and 4 black; black setulose. Genitalia as in Fig. 132.

#### Notes.

The studied female from Sabah (Malaysia) possibly belongs to a different species. Usually in Microdontinae, females are wider in body dimensions than males. In this specimen the face and tergite 2 are narrower than in the male holotype. This may indicate that it belongs to a different species. This matter is here left unresolved, because so few specimens are available.

#### Distribution.

Known from Peninsular Malaysia and possibly Sabah.

### ﻿ Paramixogaster sacki

Taxon classificationAnimaliaDipteraSyrphidae

Reemer & Ståhls, 2013

985BBDA4-17D4-533B-848F-B75ABC48B5F6

Figs 21 , 95–101 , 133

Myxogaster variegata Sack, 1922: 274. Syntypes ♀: Taiwan (type lost?) [not examined].
Paramixogasteroides variegata (Sack, 1922) – Shiraki 1930: 9; Knutson et al. 1975: 374.
Paramixogaster variegata (Sack, 1922) – Reemer and Ståhls 2013a: 145.
Paramixogaster sacki Reemer & Ståhls, 2013a: 145.

#### Type specimens.

***Syntypes*** of *Myxogastervariegata* Sack. Taiwan • 2 ♀; Toa Tsui Kutsu; lost [not examined]. ***Neotype*** of *Myxogastervariegata* Sack (new neotype designation, see notes). Taiwan • 1 ♂; Nantou Lienhuachih watershed no. 3; 5 Jun. – 24 Jul. 2006 // C.S. Lin & W.T. Yang leg.; Malaise trap; RMNH. Label 1: “Taiwan Nantou // Lienhuachih // Watershed No: 3 // VI/5–VII/24/2006 // C.S. Lin & W.T. Yang // Malaise trap (KCN)”; label 2: “Paramixogaster // sacki // Det. M. Reemer 2022 // Specimen code MR1391”; label 3 (red): “NEOTYPE // *Myxogastervariegata* // Sack, 1922 // Designated by Reemer & // Sankararaman 2024”.

#### Additional specimens.

Taiwan • 1 ♂; Nantou, Yuanfeng; 13 Aug. – 10 Sep. 2002; C.S. Lin & W.T. Yang leg.; Malaise trap; RMNH • 1 ♂; Nantou, Lienhuachih Watershed No: 3; 5 Jun. – 24 Jul. 2006; C.S. Lin & W.T. Yang leg.; Malaise trap; RMNH.

#### Diagnosis.

Body length: male 9–12 mm (*n* = 3); female 10 mm (Sack 1922). This species differs from all other known species of *Paramixogaster* by its unique colour pattern: the mesoscutum is largely yellow, with small two black maculae posterior to the postpronotum and two elongate narrow black maculae between the transverse suture and the posterior margin (Fig. 21). Tergites 3 and 4 are yellow with a unique pattern of black vittae (Fig. 95). Male genitalia as in Fig. 133.

#### Notes.

Sack (1922) described *Myxogastervariegata* based on two female syntypes. Attempts to locate the types at the German institutions DEI and ZMHU were unsuccessful (Eliana Buenaventura, Sven Marotzke, Frank Menzel and Joachim Ziegler pers. comm. 2020). The syntypes are considered to be lost. However, the description and figure by Sack (1922) provide enough information to identify the studied specimens as this species. We designate here a neotype to ensure the proper and consistent interpretation of the name.

Shiraki (1930) found out that this species is not closely related to the New World genus *Mixogaster* Macquart, 1842 and erected a new genus for it: *Paramixogasteroides* Shiraki, 1930. He also gave a redescription of the species based on a male and a female. *Paramixogasteroides* was synonymised with *Paramixogaster* by Cheng and Thompson (2008), which was followed by Reemer and Ståhls (2013a) and in this paper.

#### Distribution.

Taiwan.

### ﻿ Paramixogaster sulawesiana

Taxon classificationAnimaliaDipteraSyrphidae

Reemer sp. nov.

89D80A0F-14F1-5C5D-ACA0-46082E20F8D5

https://zoobank.org/CA474375-C39E-4B84-A415-AFE8516003C2

Figs 12 , 102–108 , 134

#### Type material.

***Holotype*.** Indonesia • 1 ♂; Sulawesi, Rantepao; July 1936; leg. L.J. toxopeus; coll. RMNH. Label 1: “C. [handwritten, printed letters SW are crossed out] Celebes // VII // Rantepao. // L.J. Toxopeus 1936”; label 2: “Paramixogaster // sp. n. // Det. M. Reemer 2021 // Specimen MR1392”; label 3 (red): “HOLOTYPE // Paramixogaster // sulawesiana // Reemer 2024”.

***Paratypes*.** Indonesia • 1 ♂ 1 ♀; Sulawesi, coll. USNM. [specimen labels only state “Macassar”; [unique specimen identifiers USNMENT01541879 and USNMENT01541880 for male and female, respectively].

#### Diagnosis.

Body length: 8–9.5 mm. Belongs to the group of species with lateral bulges on the frons (Figs 104, 105, 107). From *P.fujianensis* it differs by tergite 2 being less than twice as long as wide (more than twice as long as wide in *P.fujianensis*). From *P.icariiformis* it differs by the presence of fasciae of golden setulae along the posterior margins of tergites 3 and 4 (absent in *P.icariiformis*). There is a continuous fascia of golden setulae along the transverse suture of the scutum, and there are fasciae of golden setulae along the posterior margins of tergites 3 and 4 (Fig. 102). These fasciae are not as dense and as sharply demarcated as in *P.kodaiana* Sankararaman & Reemer, sp. nov. (from which it also differs by the lack of dark colouration in wing cell r_4+5_), but more similar to those in *P.huoi* Reemer, nom. nov. From the latter species, *P.sulawesiana* Reemer, sp. nov. differs by the wing venation: the apex of R_2+3_ is situated well beyond the joint of M_1_ with R_4+5_ (at approximately the same level in *P.huoi* Reemer, nom. nov.). Male genitalia as in Fig. 134.

#### Description

**(based on holotype). Adult male.** Body length: 9 mm.

***Head*.** Face occupying ~ 1/2 of head width in frontal view, with sides somewhat converging ventrad; blackish brown with widely yellow lateral and ventral margins; entirely golden yellow setulose. Gena yellow, yellow setulose. Oral margin not notched anteriorly, laterally weakly produced. Frons posteriorly with shining blackish pair of lateral bulges which are short black setulose, except golden yellow setulose on a triangular patch adjacent to eye margin, these bulges are separated by a narrow yellowish crease; frons anteriorly (laterad of antennal fossa) with pair of more or less flat yellow areas which are golden yellow setulose, separated from face by pair of shiny black bare maculae. Vertex swollen, with oblique depressions converging anteriad; blackish brown; short black setulose except golden yellow setulose along all margins. Occiput black; golden yellow setulose dorsally, white setulose ventrally. Eye bare. Antennal fossa approximately as high as wide. Antenna orange-brown; postpedicel 6.5× as long as scape. Arista ~ 2× as long as scape.

***Thorax*.** Mesoscutum blackish brown; short black setulose, except for narrow fascia of golden yellow setulae along transverse suture, large patch of golden yellow setulae anterior to scutellum, and small patch of golden yellow setulae anterior to postalar callus. Postpronotum brown, bare. Postalar callus yellow, golden yellow setulose. Scutellum without calcars; yellow; golden yellow setulose. Pleura yellowish dorsally, brown ventrally. Anepisternum and anepimeron entirely covered with thick golden yellow setulae, appressed and directed hindward. Katepisternum long golden yellow setulose dorsally; bare ventrally. Katepimeron with a few long yellow setulae. Katatergite and anatergite short microtrichose. Metanotum shining brown. Calypter and halter yellow.

Wing: hyaline; microtrichose, except bare in cell r_1_ narrowly along Rs, narrowly along veins in basal 1/3 of r_4+5_, entirely on cell br (except for microtrichia along vena spuria), narrowly along veins in antero-basal 1/5 of cell dm, entirely on cell bm, basal 2/3 of cell cup.

Legs: yellowish brown, except hind femur darker brown.

***Abdomen*.** Constricted basally, narrowest at basal 1/6 of tergite 2, widest at transition between tergites 3 and 4. Tergite 1 dark brown; yellowish setulose. Tergite 2 dark brown with two large, elongate, pale yellow maculae from anterior margin to ~ 3/5 of tergite, and yellow fascia of ~ 1/6 of tergal length along posterior margin; short black setulose, except bare on yellow maculae with fascia of thick golden yellow setulae along posterior margin. Tergite 3 dark brown, except yellowish brown fascia of ~ 1/5 of tergal length along posterior margin; black setulose anteriorly, golden yellow setulose posteriorly. Tergite 4 dark brown except widely yellow along posterior and lateral margins; short black setulose antero-medially, golden yellow setulose postero-medially and laterally. Sternite 1 brown; sparsely short black setulose. Sternite 2 yellow; bare. Sternites 3 and 4 brown; yellow setulose. Genitalia as in Fig. 134.

#### Notes.

The male paratype differs from the holotype in the following aspects: body length 8.5 mm; postpedicel 8× as long as scape; vertex golden yellow setulose; dark parts on head, thorax, and abdomen more brownish (rather than blackish as in holotype). The female paratype (in which the antennae are missing) differs from the male holotype in the same aspects of colouration, as well in the body length of 9 mm. The colour differences between holotype and paratype are considered to either represent intraspecific variation or result from differences in preservation history.

#### Distribution.

Only known from Sulawesi (Indonesia).

#### Etymology.

The specific epithet (adjective) refers to the type locality.

### ﻿ Paramixogaster vespiformis

Taxon classificationAnimaliaDipteraSyrphidae

(de Meijere, 1908)

1B6A507D-AD34-5458-9439-0F9F3E182A7D

Figs 46–51 , 52–53 , 117–122 , 137

Microdon vespiformis de Meijere, 1908: 210. Lectotype ♀: Indonesia, Java (RMNH) [examined]; Knutson et al. 1975: 372.
Paramicrodon decipiens de Meijere, 1917: 242. Holotype ♀: Indonesia, Java (RMNH) [examined].
Paramicrodon dicipiens de Meijere, 1917 – Knutson et al. 1975: 373 (misspelling).
Paramixogaster decipiens (de Meijere, 1917) – Reemer and Ståhls 2013a: 145.
Paramixogaster vespiformis (de Meijere, 1908) – Reemer and Ståhls 2013a: 145.

#### Studied type specimens.

***Lectotype*** of *Microdonvespiformis* de Meijere (designated here, see notes). Indonesia • 1 ♀; label 1: “E. Jacobson Batavia Sept. 1907”; label 2: “*Microdonvespiformis* type det. de Meijere”; label 3 (red): “*Microdonvespiformis* de Meijere, 1908 ZMAN type DIPT.1074.1”; RMNH.

Indonesia • 1 ♀, paralectotype (new designation, see notes) of *Microdonvespiformis* de Meijere; label 1: “*Microdonvespiformis*”; label 2: “*Microdonvespiformis* de Meijere, 1908 ZMAN type? DIPT.1074”; RMNH.

***Holotype*** of *Paramicrodondecipiens* de Meijere. Indonesia • 1 ♀; Java; RMNH. Label 1: “Salatiga V.1915 Roepke”; label 2: “*Paramicrodondecipiens* det. de Meijere Type”; label 3 (red): “Microdondecipiens de Meijere, 1917 ZMAN type DIPT.0975.1”; RMNH.

***Paratypes*** of *Paramicrodondecipiens* de Meijere (only puparia, no adult specimens, although probably the holotype was reared from one of these specimens). Indonesia • 3 empty puparia on a piece of dry leaf. Label 1: “Salatiga V.1915 Roepke”; label 2: “*Paramicrodondecipiens* de Meijere, 1917 ZMAN type? DIPT.0975”.

#### Additional specimens.

Indonesia • 1 ♀; Java; Apr. 1908; E. Jacobson leg.; RMNH • 1 ♀; Java, Dungus Iwul; 2 Dec. 1952; alt. 100 m; M.A. Lieftinck leg.; RMNH • 3 ♂ 1 ♀; Sumatra, Fort De Kock; alt. 920 m; 1925; E. Jacobson leg.; RMNH • 1 ♀; W. Bali, nr. Negara, rainforest above Batuagung; alt. 550 m; 4–6 Dec. 1991; C. van Achterberg leg.; RMNH.

Malaysia • 1 ♂; Penang; 1927; C.F. Baker leg.; USNM • 1 ♂; Penang; 8 Dec. 1942; H.T. Pagden leg.; NHMUK [13933416].

Philippines • 1 ♂; Palawan, Brookes, Point Uring Uring; 16 Aug. 1961; Noona Dan. Exp. 61–62 leg.; ZMUC • 1 ♂; Palawan, Brookes, Point Uring Uring; 10 Sep. 1961; Noona Dan. Exp. 61–62 leg.; ZMUC • 1 ♂; Palawan, Mantalingajan, Pinigisan; 7 Sep. 1961; Noona Dan. Exp. 61–62 leg.; ZMUC • 1 ♂; Balabac, Dalawan Bay; 8 Oct. 1961; Noona Dan. Exp. 61–62 leg.; ZMUC • 1 ♂; Balabac, Dalawan Bay; 13 Oct. 1961; Noona Dan. Exp. 61–62 leg.; ZMUC • 1 ♀; Balabac, Dalawan Bay; 10 Oct. 1961; Noona Dan. Exp. 61–62 leg.; ZMUC.

Thailand • 1 ♀; Chantaburi Prov., Tha Mai District, Ao Khating; 1 Jan. 1992; G.R. Ballmer leg.; UCRC [label: “Photo KC64-318:31-33”]

#### Diagnosis.

Body length: males 7–9 mm (*n* = 7), females 6–10 mm (*n* = 6). This belongs to the group of species without lateral bulges on the frons. From *P.luxor* it differs by the postpedicel being longer than the scape (shorter in *P.luxor*). From *P.contracta* and *P.conveniens* it differs by the incomplete transverse suture (complete in *P.contracta* and *P.conveniens*). From *P.sacki* it differs by tergites 3 and 4 being black with yellow posterior margin (yellow with pattern of black vittae in *P.sacki*). It differs from *P.jubata* Reemer, sp. nov. by the shorter tergite 2, of which the posterior margin is wider than the median length (longer in *P.jubata* Reemer, sp. nov.), the dark anterior margin of the scutellum (entirely yellow in *P.jubata* Reemer, sp. nov.) and the shorter setulae on the vertex.

*Paramixogastervespiformis* is most similar to *P.indica*, from which it differs by the interrupted yellow vitta between postpronotum and posterior callus (continuous in *P.indica*), and the shorter postpedicel in the male, which is 3.3–3.7× as long as the scape (4.4–5.6× as long in *P.indica*). Male: postpedicel 3.3–3.7× as long as scape. Female: postpedicel 1.6–2.9× as long as scape. The degree of infuscation of wing apex is very variable. Male genitalia as in Fig. 137.

#### Notes.

The description of *Microdonvespiformis* by de Meijere (1908) was based on an unknown number of specimens. The specimen identified as syntype by de Jong (2000) is clearly a primary type, based on the label information and the concurrence of its characters with the original description. This specimen is here designated as lectotype. The RMNH collection also holds a female specimen which is considered by de Jong (2000) as a possible syntype of *Microdonvespiformis* de Meijere. The label is in de Meijere’s handwriting and the specimen agrees well with the other syntype, except that it is smaller (6 mm), and it has a peculiar forked appendix on vein R_4+5_. This latter character is considered as an abnormality. Unlike the lectotype, however, this specimen has no locality information on the label. Besides, de Meijere (1908) does not mention a smaller specimen with an aberrant wing venation. Therefore, this specimen is here regarded as not belonging to the type series.

In the same paper as the one in which he described *Microdonvespiformis*, de Meijere (1908) also described specimens from Bali which he identified as *Microdonindicus* (Doleschall). However, as de Meijere noted himself, these specimens differ from *M.indicus* as described by Doleschall (1857), and also from *Microdonvespiformis* de Meijere, 1908, because the frons is rather uneven (‘ziemlich uneben’) and bears two large, round elevations (‘etwas erhabenen grossen runden Stellen’). This character reminds of the lateral bulges on the frons found in several other *Paramixogaster* species (e.g., *P.icariiformis*, *P.sulawesiana* Reemer, sp. nov., Fig. 6), but not in *P.indica* or *P.vespiformis*. Probably, the Balinese specimens referred to by de Meijere as *C.indica* were misidentified. Unfortunately, no specimens identified by de Meijere as *C.indica* could be found in the collection of the RMNH (which nowadays includes the collection of the former ZMAN, in which most of de Meijere’s material was deposited). So, the identity of *Microdonindicus* (Doleschall) *sensu*de Meijere (1908) remains unclear.

The empty puparia (Figs 52, 53) listed among the type specimens of *Paramicrodondecipiens* de Meijere have also been described by de Meijere (1917), so these can be regarded to belong to the type series (de Jong 2000). As the species description is based on the single adult female, thus the holotype, the empty puparia are considered paratypes.

In the holotype of *P.decipiens* there is no appendix on vein R_4+5_. Otherwise, the species is very similar to the other specimens here identified as *P.vespiformis*. In some of the specimens from the Philippines this appendix is also lacking, whereas in one specimen it is only present in one of the wings.

*Paramixogastervespiformis* is very similar to *P.indica*, so such an extent that these taxa might be considered synonymous as well in the future. Unfortunately, most of the available specimens are at least several decades old, so molecular analyses are not very feasible. As the morphological differences are small, but consistent, here the view is taken that these taxa represent two different, albeit closely similar species.

The separation between the ranges of *P.indica* and *P.vespiformis* seems to follow the line of Wallace, as well as Huxley’s adaptation of it (Lohman et al. 2011), with *P.indica* being the Wallacean species and *P.vespiformis* occurring on the Sunda Shelf. The single exception seems to be a female specimen from Bali (collected near Negara rainforest, above Batuagung, 4–6.XII.1991, leg. C. van Achterberg, coll. RMNH). The yellow lateral vitta along the scutum is continuous in this specimen, which would indicate *P.indica*. However, the other characters differentiating between *P.indica* and *P.vespiformis* can only be seen in males, so identification based on this single colour character remains a bit uncertain. This female specimen is here left unidentified and it is therefore not listed among the studied specimens. It would not be the first ‘Wallacean’ taxon to colonise Bali (Tänzler et al. 2014), but more specimens are needed to confirm that this is indeed the case.

A larva of this species was found by Greg R. Ballmer (pers. comm. 2023) in Thailand in 1992 in a folded leaf shelter, also occupied by ants, putative *Dolichoderusthoracicus* (Smith, 1860) (Fig. 4). This specimen was reared to the adult stage, and the adult specimen is mounted together with the empty puparium. See section *Additional material* for further details.

#### Distribution.

Known from Thailand, Peninsular Malaysia, the Indonesian islands Sumatra, Java and Bali, and the Philippines. From the Philippines, all specimens are from the islands Balabac and Palawan. All known localities are situated west of the Wallace Line (and also of Huxley’s adaptation of it).

### ﻿ Paramixogaster yunnanensis

Taxon classificationAnimaliaDipteraSyrphidae

Cheng in Huang & Cheng, 2012

444EEAAB-05BD-5442-8C0C-3DC18E298BB7

Paramixogaster yunnanensis Cheng in Huang & Cheng, 2012: 696. Holotype ♂: China, Yunnan (CASB, but see notes) [not examined]; Reemer and Ståhls 2013a: 145.

#### Diagnosis.

Only male known. Body length: 7 mm. This belongs to the group of species with lateral bulges on the frons. From *P.fujianensis* it differs in tergite 2 being less than twice as long as wide (more than twice as long as wide in *P.fujianensis*). From *P.icariiformis*, *P.kodaiana* Sankararaman & Reemer, sp. nov., *P.huoi* Reemer, nom. nov. and *P.sulawesiana* Reemer, sp. nov. it differs by the absence of a fascia of golden setulae along the transverse suture of the scutum (present in the four aforementioned species). From *P.brunettii* it differs by the black tergite 2 (reddish in *P.brunettii*) with a pair of yellow maculae. From *P.halmaherensis* Reemer, sp. nov. it differs by the dark postalar calli (yellow in *P.halmaherensis* Reemer, sp. nov.), the longer postpedicel, which is 8× as long as scape (6× as long in *P.halmaherensis* Reemer, sp. nov.), and the entirely clear wing (infuscate in apical 1/2 of cells r_1_ and r_2+3_ in *P.halmaherensis* Reemer, sp. nov.). Figures of habitus and head are provided by Huang and Cheng (2012). Note that these characters are based on the description only and could not be verified against any specimens.

#### Notes.

Unsuccessful attempts were made to locate the type specimen of *Paramixogasteryunnanensis* by trying to contact the author and by enquiring at the CASB collection (Ke-Ke Huo pers. comm. 2023). The original description in Huang and Cheng (2012) is in Chinese, but the same work also provides an English translation, as well as figures of the head in frontal view and of the thorax and abdomen in dorsal view. This information suggests that *P.yunnanensis* is very similar to *P.halmaherensis* Reemer, sp. nov.

**Figures 29–31.**
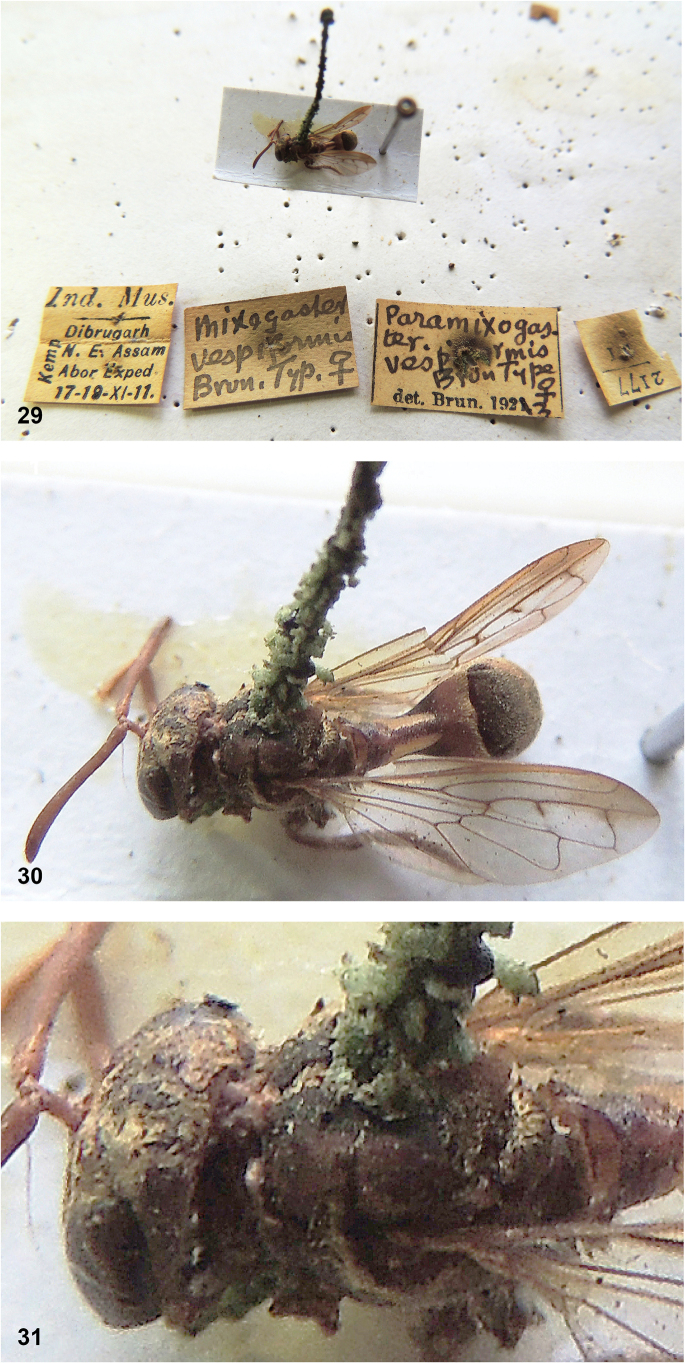
*Paramixogasterbrunettii* Reemer, holotype: **29** mounted specimen and labels **30** habitus dorsolateral **31** head and thorax dorsolateral. Photos by J. van Steenis.

**Figures 32–38.**
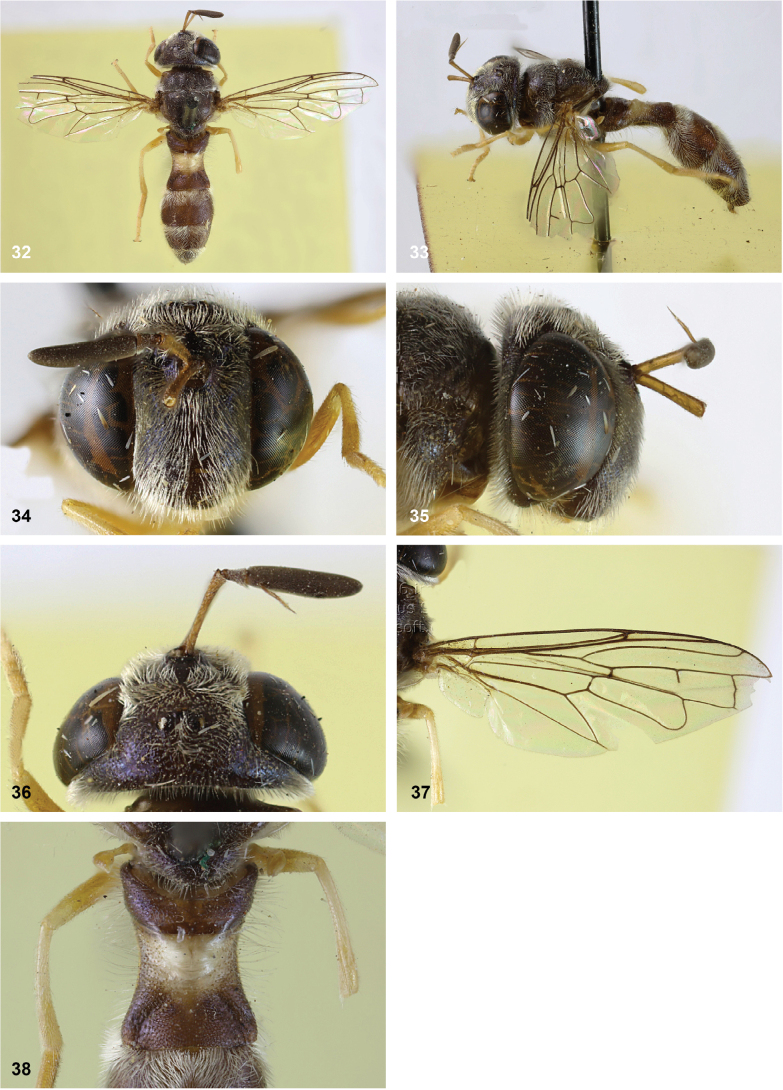
*Paramixogastercontracta* (Brunetti), female, holotype: **32** habitus, dorsal **33** habitus, lateral **34** head, frontal **35** head, lateral **36** head, dorsal **37** wing **38** tergite 2, dorsal.

**Figures 39–45.**
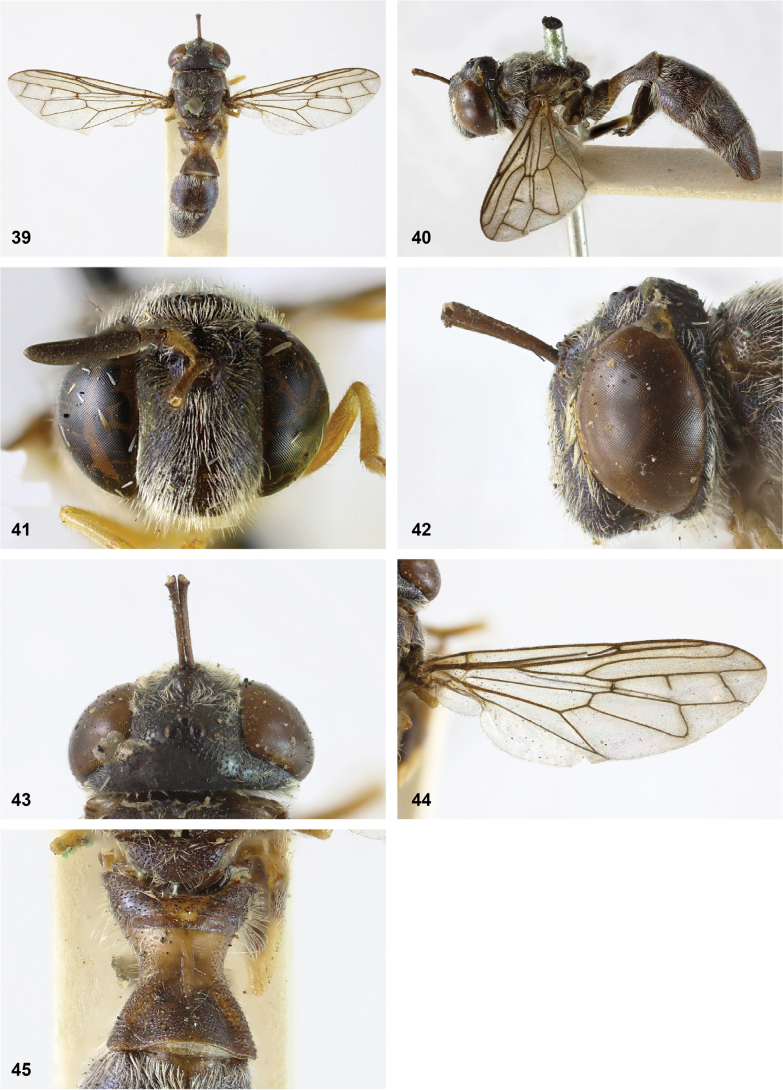
*Microdonconveniens* Brunetti, female, holotype: **39** habitus, dorsal **40** habitus, lateral **41** head, frontal **42** head, lateral **43** head, dorsal **44** wing **45** tergite 2, dorsal.

**Figures 46–51.**
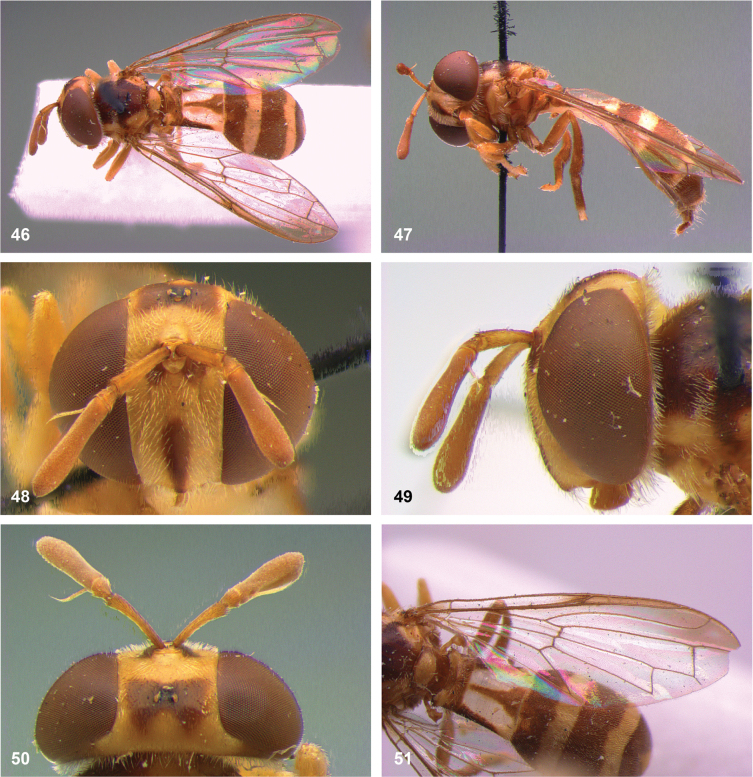
*Microdondecipiens* de Meijere, female, holotype: **46** habitus, dorsal **47** habitus, lateral **48** head, frontal **49** head, lateral **50** head, dorsal **51** wing.

**Figures 52, 53.**
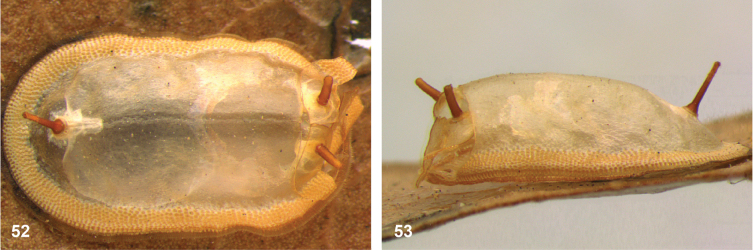
*Microdondecipiens* de Meijere, puparium, paratype: **52** habitus, dorsal **53** habitus, lateral.

**Figures 54–59.**
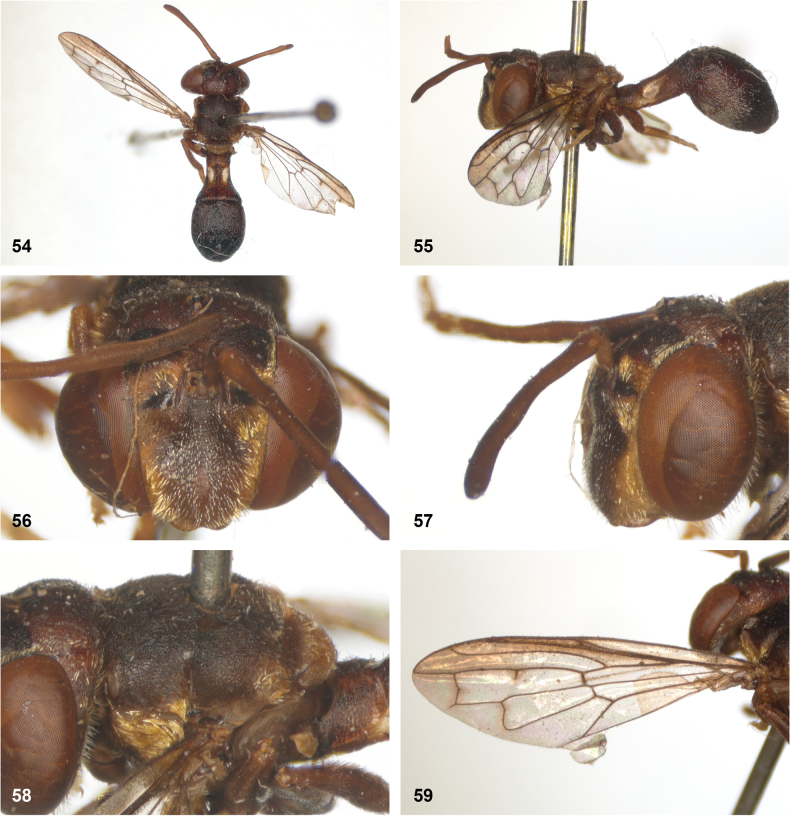
*Paramixogasterhalmaherensis* Reemer, sp. nov. male, holotype: **54** habitus, dorsal **55** habitus, lateral **56** head, frontal **57** head, fronto-lateral **58** thorax, dorso-lateral **59** wing.

**Figures 60–66.**
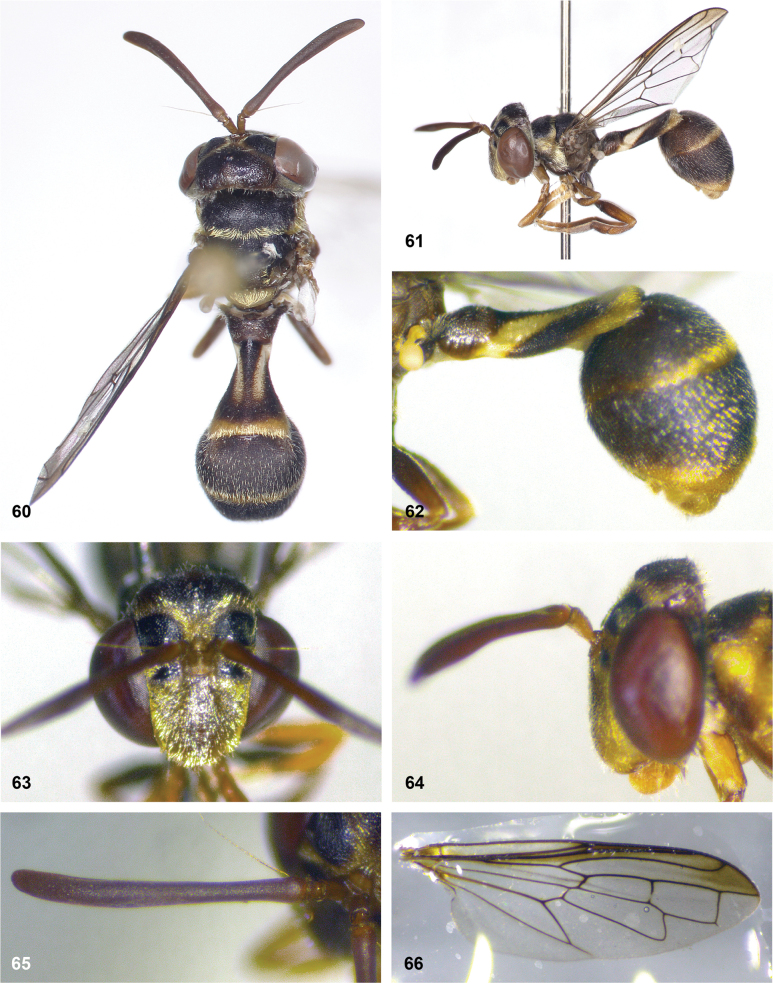
*Paramixogasterhuoi* Reemer, nom. nov. male, holotype: **60** habitus, dorsal **61** habitus, lateral **62** abdomen, lateral **63** head, frontal **64** head, lateral **65** antenna **66** wing.

**Figures 67–72.**
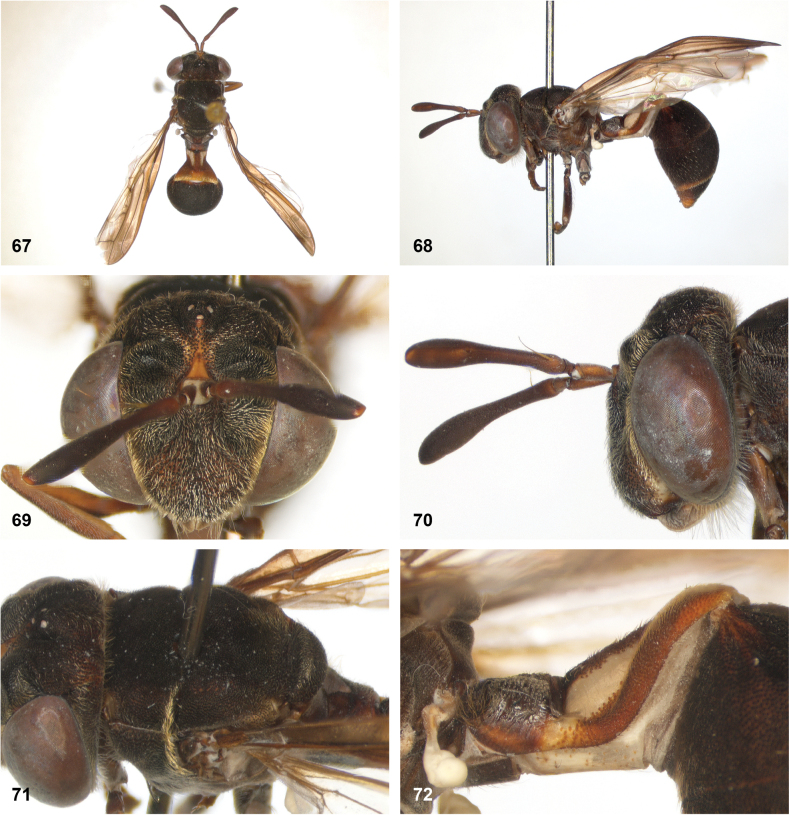
*Paramixogastericariiformis* Pendlebury female, neotype: **67** habitus, dorsal **68** habitus, lateral **69** head, frontal **70** head, lateral **71** thorax, dorso-lateral **72** tergite 2, lateral.

**Figures 73–80.**
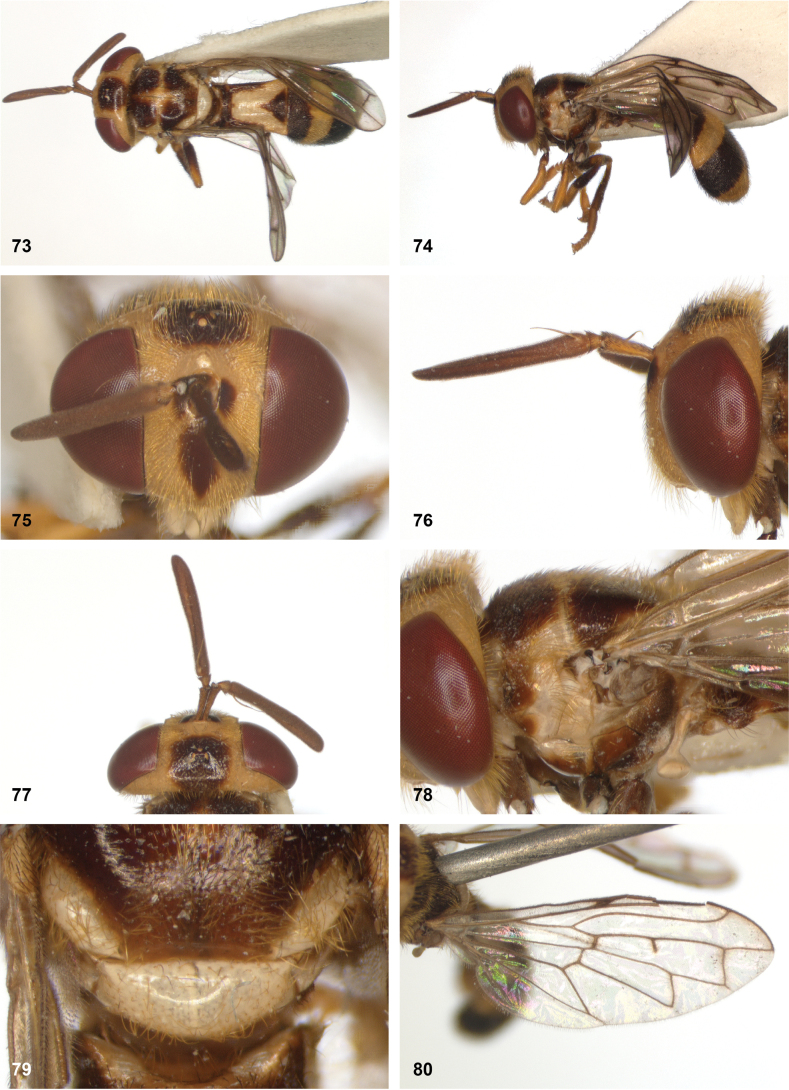
*Paramixogasterjubata* sp. nov. male, holotype: **73** habitus, dorsal **74** habitus, lateral **75** head, frontal **76** head, lateral **77** head, dorsal **78** thorax, lateral **79** scutellum, dorsal **80** wing.

**Figures 81–87.**
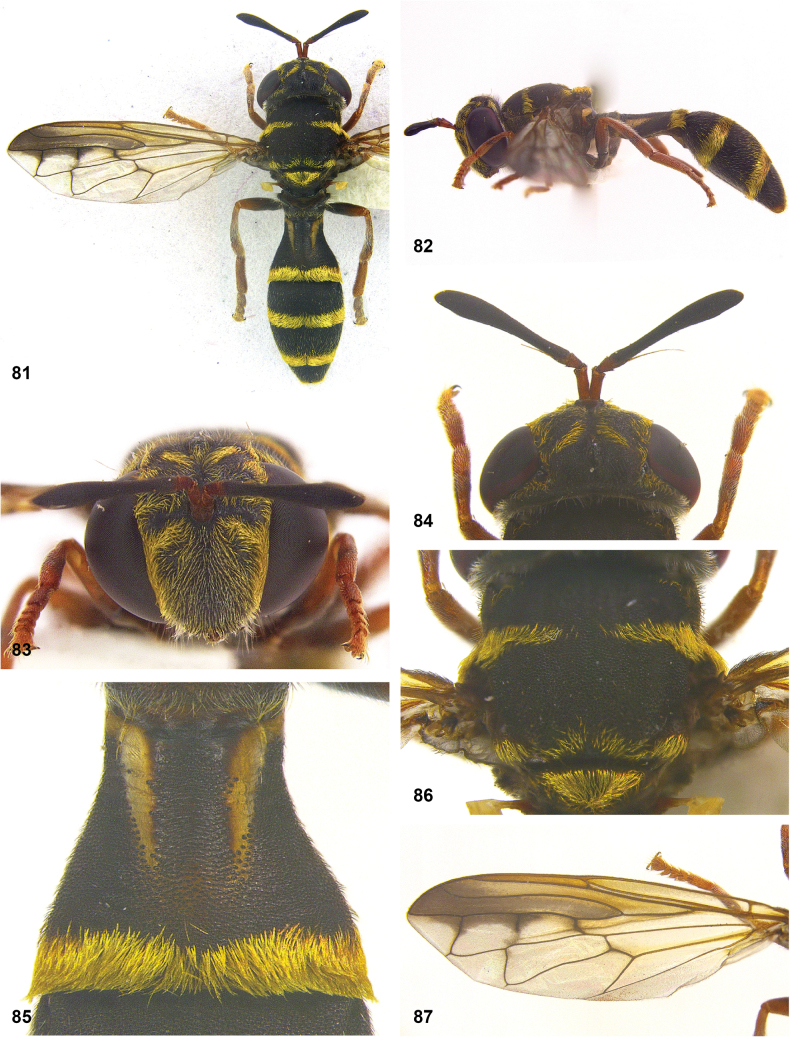
*Paramixogasterkodaiana* Sankararaman & Reemer, sp. nov. female, holotype: **81** habitus, dorsal **82** habitus, lateral **83** head, frontal **84** head, dorsal **85** tergite 2, dorsal **86** thorax, dorsal **87** wing.

**Figures 88–90.**
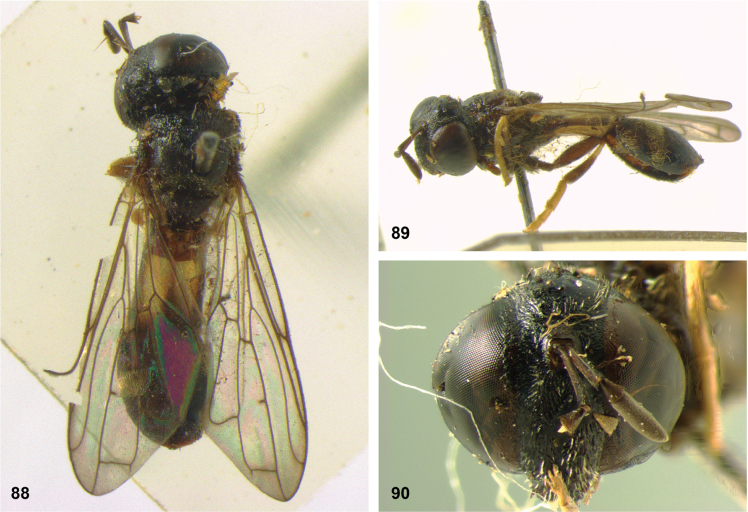
*Microdonluxor* Curran male, holotype: **88** habitus, dorsal **89** habitus, lateral **90** head, frontal.

**Figures 91–94.**
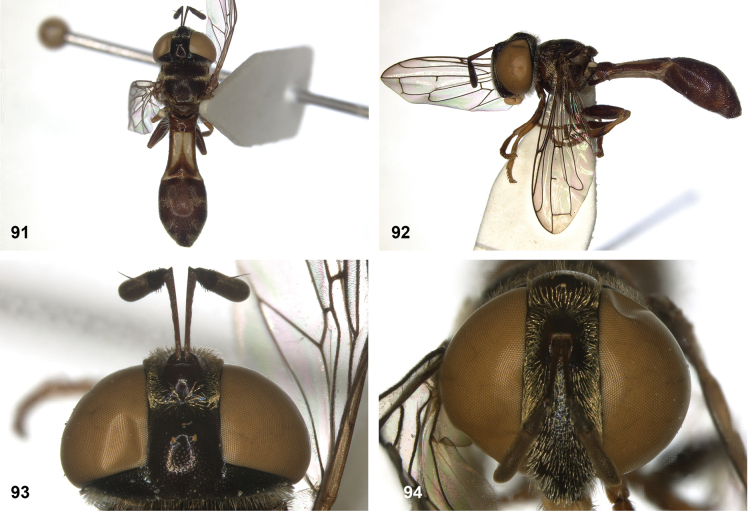
Paramixogastercf.luxor (Curran) female, Sabah: **91** habitus, dorsal **92** habitus, lateral **93** head, dorsal **94** head, frontal.

**Figures 95–101.**
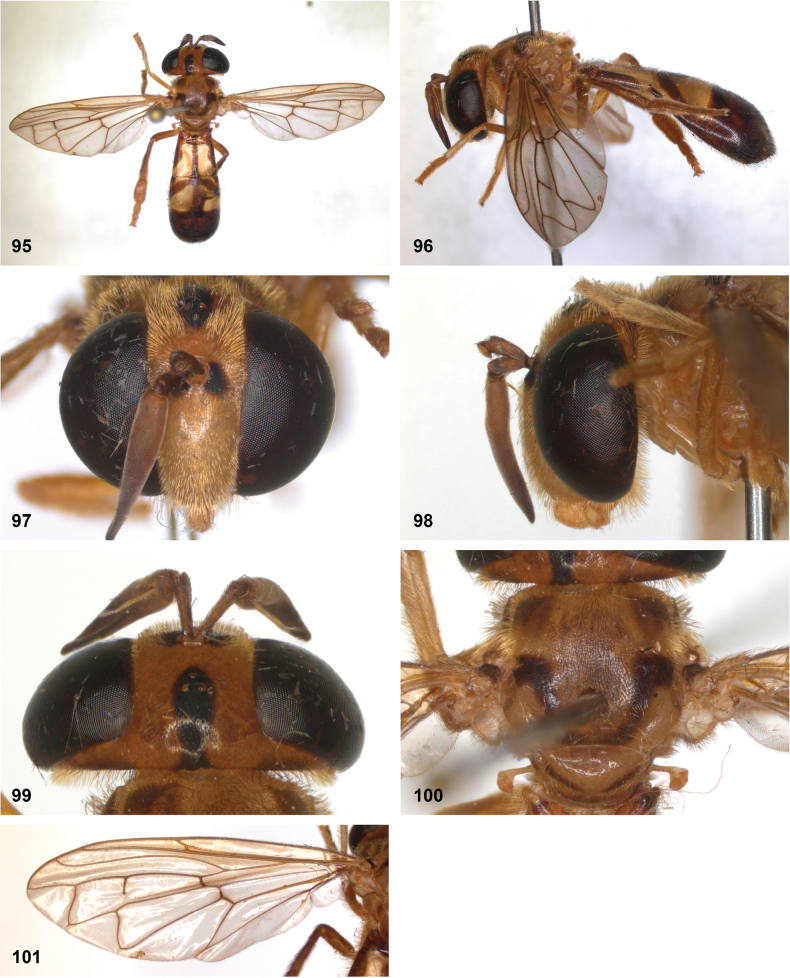
*Paramixogastersacki* Reemer & Ståhls male, neotype: **95** habitus, dorsal **96** habitus, lateral **97** head, frontal **98** head, lateral **99** head, dorsal **100** thorax, dorsal **101** wing.

**Figures 102–108.**
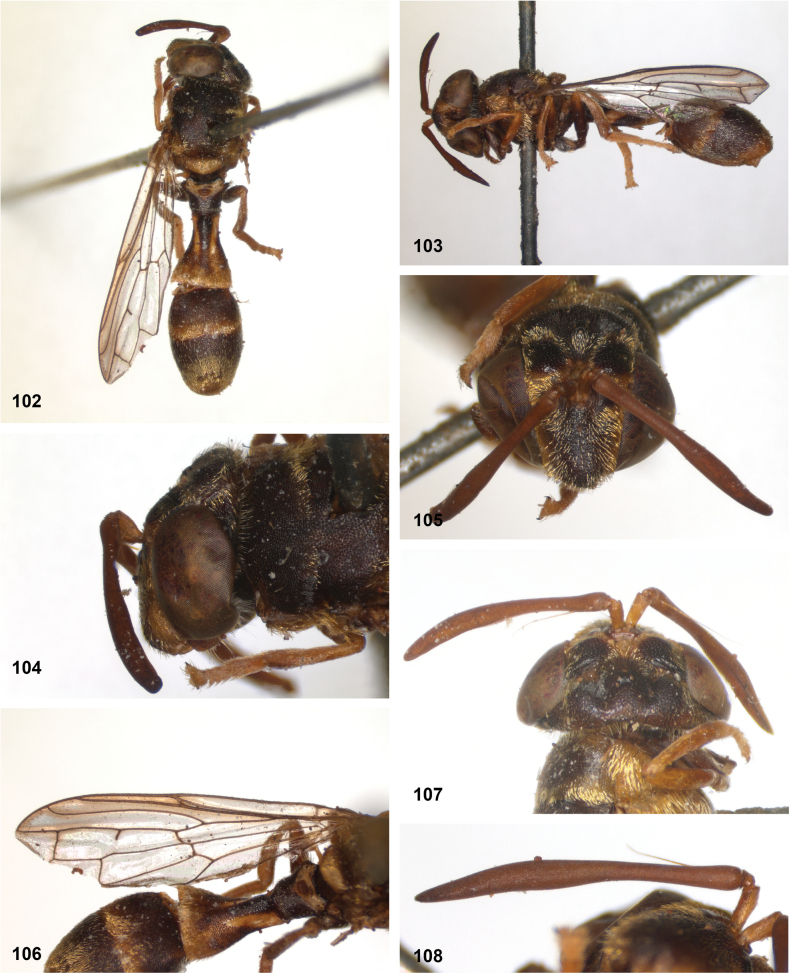
*Paramixogastersulawesiana* sp. nov. male, holotype: **102** habitus, dorsal **103** habitus, lateral **104** head, lateral **105** head, frontal **106** wing **107** head, dorsal **108** antenna.

**Figures 109–113.**
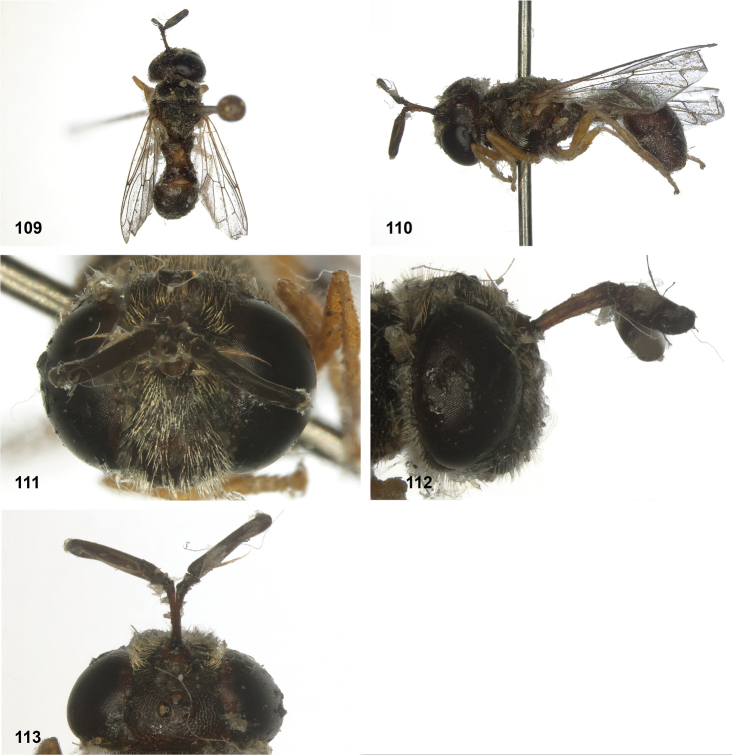
*Microdonsubpetiolatus* Thompson male, holotype: **109** habitus, dorsal **110** habitus, lateral **111** head, frontal **112** head, lateral **113** head, dorsal.

**Figures 114–116.**
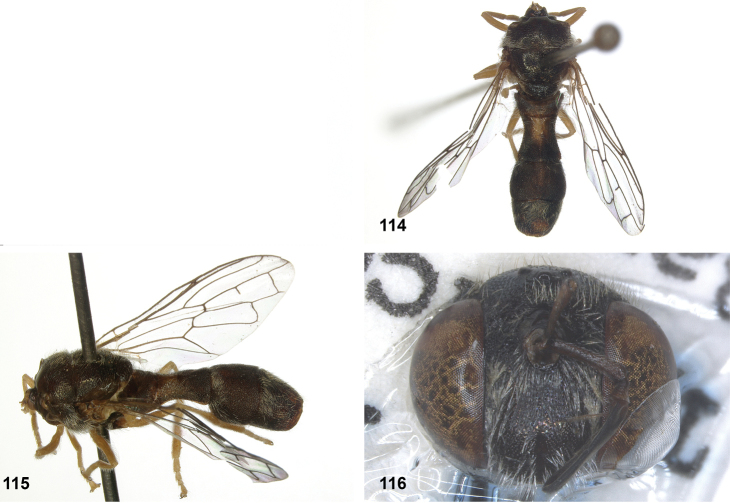
*Microdonsubpetiolatus* Thompson male, paratype: **114** habitus, dorsal **115** habitus, dorso-lateral **116** head, frontal.

**Figures 117–122.**
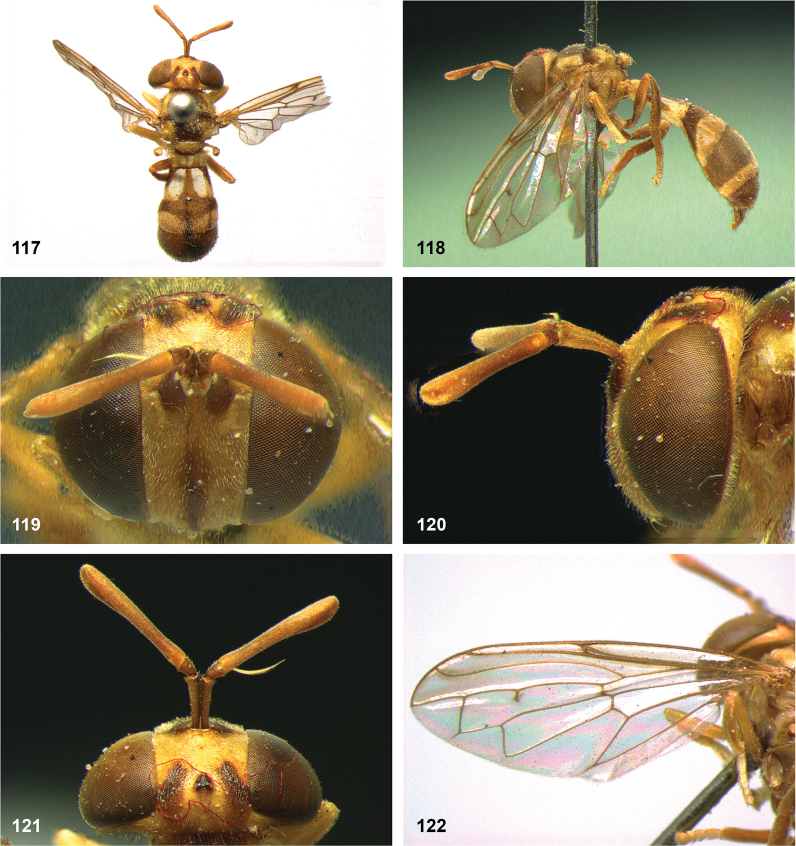
*Microdonvespiformis* de Meijere female, lectotype: **117** habitus, dorsal **118** habitus, lateral **119** head, frontal **120** head, lateral **121** head, dorsal **122** wing.

**Figures 123, 124.**
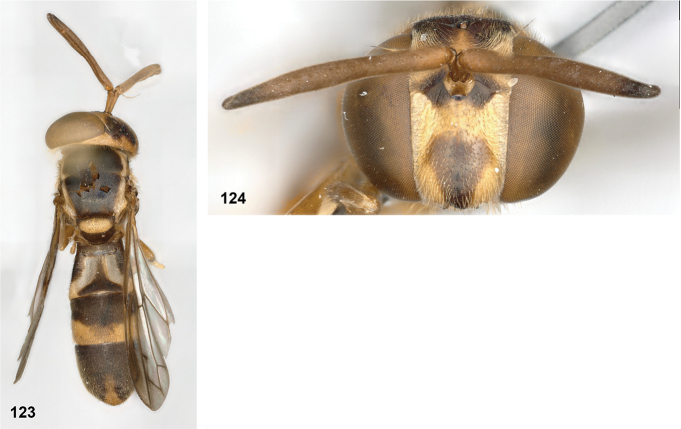
*Paramixogasterwegneri* Keiser male, holotype: **123** habitus, dorsal **124** head, frontal.

**Figures 125–129.**
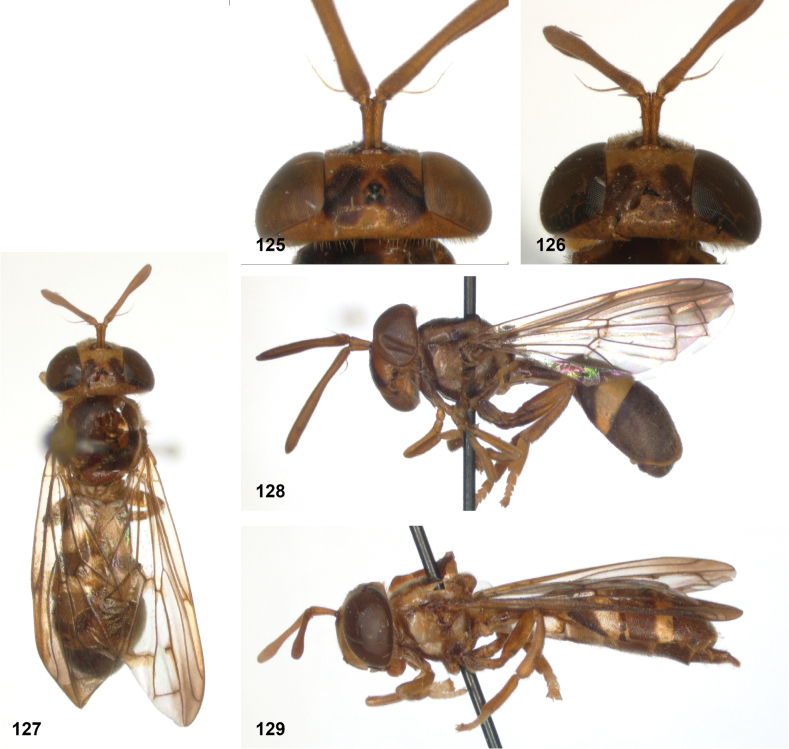
*Paramixogasterwegneri* Keiser, paratypes: **125** head, dorsal, male **126** head, dorsal, female **127** habitus, dorsal, female **128** habitus, lateral, male **129** habitus, lateral, female.

**Figures 130–137.**
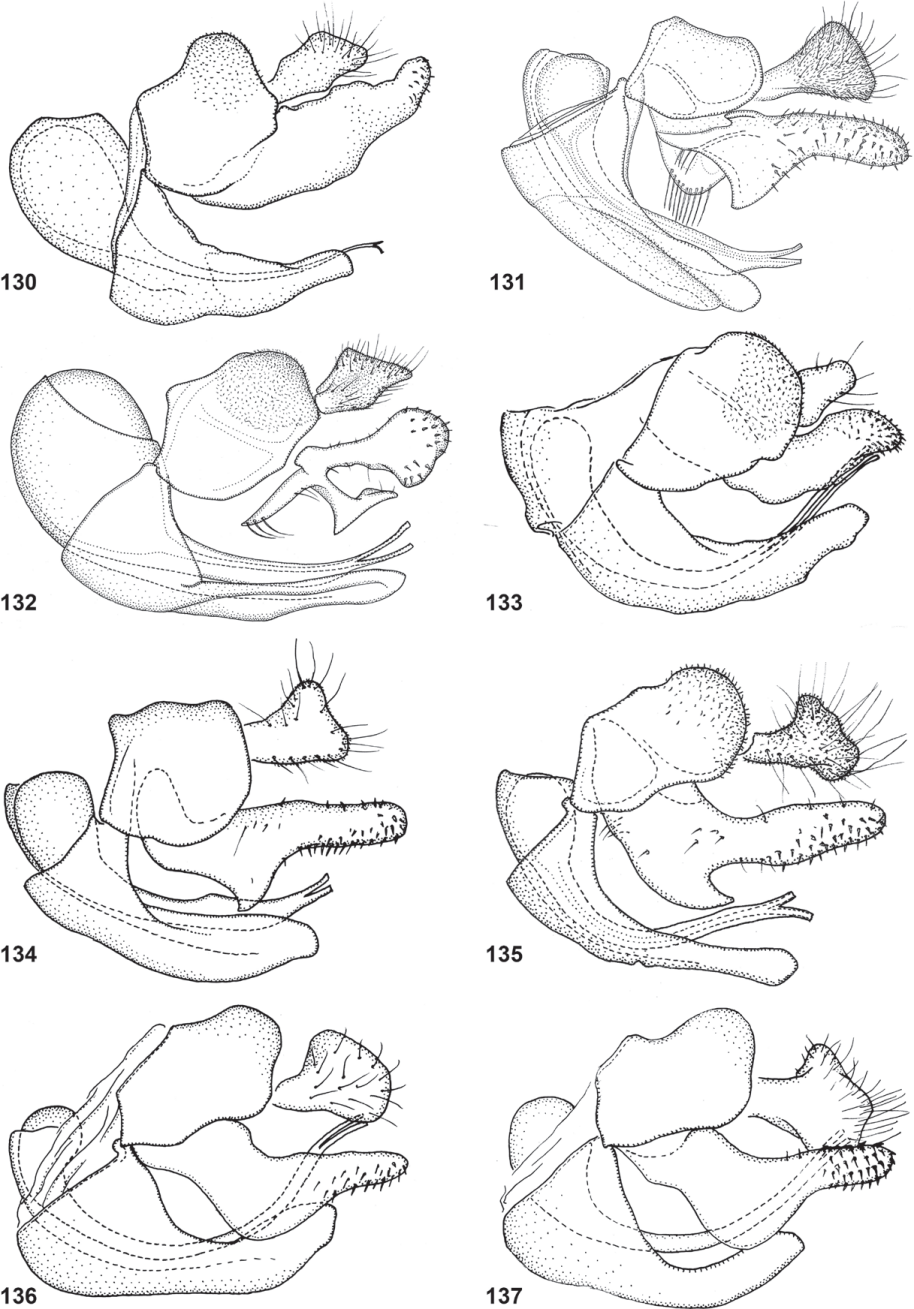
Male genitalia of *Paramixogaster* species: **130***P.contracta* (paratype *Microdonsubpetiolatus* Thompson) **131***P.halmaherensis* Reemer, sp. nov. holotype **132***P.luxor* holotype **133***P.sacki* Taiwan, RMNH**134***P.sulawesiana* Reemer, sp. nov. holotype **135***P.jubata* Reemer, sp. nov. paratype Vietnam **136***P.indica* (paratype *P.wegneri* Keiser syn. nov.) **137***P.vespiformis* Sumatra, RMNH.

## ﻿Discussion

For several species of *Paramixogaster* treated in this paper, the taxonomy presented here is not fully satisfying. The main cause for this is the small number of specimens available for most of the species. For nine of the 15 known Oriental species, only the type specimen is known and for another two only one sex is known. For two species (*P.fujianensis* and *P.yunnanensis*) only line drawings of the types could be studied, and for two other ones (*P.brunettii* and *P.huoi* Reemer, nom. nov.) only photographs of the types were studied. This leads to uncertainties in the key and diagnoses. Moreover, the photos in Figs 1–3 demonstrate that there is at least one additional, yet undescribed, Oriental species of *Paramixogaster*. Therefore, we advise caution when using the key and diagnoses, and to always compare specimens carefully with the descriptions and photographs.

*Paramixogasterluxor* is quite aberrant from the other Oriental species in the presence of scutellar calcars and the short postpedicel (shorter than scape). The surstylus of the male genitalia is also very different from other species in the genus because it is divided into three processes (Fig. 131). Because of these characters, it seems possible that *P.luxor* does not belong in *Paramixogaster*. Analysis of molecular characters could be helpful in recovering the phylogenetic affinities of this species, but so far these are not available.

## Supplementary Material

XML Treatment for Paramixogaster

XML Treatment for Paramixogaster brunettii

XML Treatment for Paramixogaster contracta

XML Treatment for Paramixogaster conveniens

XML Treatment for Paramixogaster fujianensis

XML Treatment for Paramixogaster halmaherensis

XML Treatment for Paramixogaster huoi

XML Treatment for Paramixogaster icariiformis

XML Treatment for Paramixogaster indica

XML Treatment for Paramixogaster jubata

XML Treatment for Paramixogaster kodaiana

XML Treatment for Paramixogaster luxor

XML Treatment for Paramixogaster sacki

XML Treatment for Paramixogaster sulawesiana

XML Treatment for Paramixogaster vespiformis

XML Treatment for Paramixogaster yunnanensis

## Footnotes

Note: an undescribed species from Malaysia, only known from photographs, also keys out here. See Figs 1–3 for photos and a brief diagnosis.

## ﻿Additional information

### Author ORCIDs

Hariharakrishnan Sankararaman https://orcid.org/0000-0002-5244-9833

### Data availability

All of the data that support the findings of this study are available in the main text.
